# Targeting Cancer Stem Cells with Phytochemicals: Molecular Mechanisms and Therapeutic Potential

**DOI:** 10.3390/biomedicines14010215

**Published:** 2026-01-19

**Authors:** Ashok Kumar Sah, Joy Das, Abdulkhakov Ikhtiyor Umarovich, Shagun Agarwal, Pranav Kumar Prabhakar, Ankur Vashishtha, Rabab H. Elshaikh, Ranjay Kumar Choudhary, Ayman Hussein Alfeel

**Affiliations:** 1 Department of Medical Laboratory Sciences, College of Applied and Health Sciences, A’ Sharqiyah University, Ibra 400, Oman; rabab.mahmoud@asu.edu.om; 2 School of Pharmaceutical Sciences, Lovely Professional University, Phagwara 144411, Punjab, India; 3 Department of Faculty and Hospital Therapy, Bukhara State Medical Institute, Bukhara 200100, Uzbekistan; mironsho@rambler.ru; 4 School of Allied Health Sciences, Galgotias University, Greater Noida 203201, Uttar Pradesh, India; shagunmpt@gmail.com; 5 Department of Biotechnology, School of Engineering and Technology, Nagaland University, Meriema, Kohima 797004, Nagaland, India; prabhakar.iitm@gmail.com; 6 Department of Medical Laboratory Technology, School of Allied Health Sciences, Sharda University, Greater Noida 201310, Uttar Pradesh, India; ankur.vashishtha@sharda.ac.in; 7 Medical Laboratory Sciences Department, College of Health and Applied Sciences, Gulf Medical University, Ajman 4184, United Arab Emirates; dr.aymanhussein@gmc.ac.ae

**Keywords:** cancer stem cells, phytochemicals, medicinal plants, stemness pathways, chemoresistance, anticancer activity

## Abstract

Cancer stem cells (CSCs) represent a small but highly resilient tumor subpopulation responsible for sustained growth, metastasis, therapeutic resistance, and recurrence. Their survival is supported by aberrant activation of developmental and inflammatory pathways, including Wnt/β-catenin, Notch, Hedgehog, PI3K/Akt/mTOR, STAT3, and NF-κB, as well as epithelial–mesenchymal transition (EMT) programs and niche-driven cues. Increasing evidence shows that phytochemicals, naturally occurring bioactive compounds from medicinal plants, can disrupt these networks through multi-targeted mechanisms. This review synthesizes current findings on prominent phytochemicals such as curcumin, sulforaphane, resveratrol, EGCG, genistein, quercetin, parthenolide, berberine, and withaferin A. Collectively, these compounds suppress CSC self-renewal, reduce sphere-forming capacity, diminish ALDH^+^ and CD44^+^/CD24^−^ fractions, reverse EMT features, and interfere with key transcriptional regulators that maintain stemness. Many phytochemicals also sensitize CSCs to chemotherapeutic agents by downregulating drug-efflux transporters (e.g., ABCB1, ABCG2) and lowering survival thresholds, resulting in enhanced apoptosis and reduced tumor-initiating potential. This review further highlights the translational challenges associated with poor solubility, rapid metabolism, and limited bioavailability of free phytochemicals. Emerging nanotechnology-based delivery systems, including polymeric nanoparticles, lipid carriers, hybrid nanocapsules, and ligand-targeted formulations, show promise in improving stability, tumor accumulation, and CSC-specific targeting. These nanoformulations consistently enhance intracellular uptake and amplify anti-CSC effects in preclinical models. Overall, the consolidated evidence supports phytochemicals as potent modulators of CSC biology and underscores the need for optimized delivery strategies and evidence-based combination regimens to achieve meaningful clinical benefit.

## 1. Introduction

Cancer continues to represent a major global health burden, accounting for substantial rates of illness and death across diverse malignancies, with incidence rising in many cancer types [1]. Cancer stem cells (CSCs) constitute a functionally distinct subset of tumor cells that possess long-term self-renewal capacity, the ability to generate diverse cancer cell populations, and the competence to initiate and maintain tumor growth. Owing to these properties, CSCs play a central role in tumor progression and are closely associated with resistance to conventional therapies, thereby driving disease relapse and metastatic spread and positioning them as critical targets for next-generation cancer therapeutics. Accumulating evidence from cancer research demonstrates that CSCs are not mere biological curiosities but key drivers of therapeutic failure: they frequently survive chemotherapy, radiotherapy, and targeted therapy, contributing directly to minimal residual disease, treatment resistance, and eventual tumor recurrence across many cancer types. This therapy-driven survival of CSCs underlies one of the most persistent problems in oncology, persistent disease and relapse despite initial tumor shrinkage, because CSCs can initiate new tumor growth and metastasis long after the bulk tumor has been reduced by standard treatments [2,3,4,5]. Recent advances in CSC biology reveal that their maintenance and survival depend on a tightly regulated network of developmental signaling pathways, notably Wnt/β-catenin, Notch, Hedgehog, PI3K/Akt/mTOR, and STAT3, as well as dynamic interactions with the tumor microenvironment [6,7,8]. These pathways not only support self-renewal but also actively mediate intrinsic therapy resistance, including increased DNA repair, quiescence, drug efflux, adaptation to microenvironmental stress, and immune evasion, which collectively protect CSCs from conventional interventions [2,9]. Consequently, effective cancer treatment increasingly hinges on strategies that eliminate both bulk tumor cells and the CSC subpopulation to achieve durable therapeutic responses [10].

Phytochemicals, naturally occurring bioactive compounds derived from plants, have received considerable attention in cancer research due to their unique properties: notably a comparatively favorable safety profile with low systemic toxicity, pleiotropic actions, and the ability to target multiple signaling cascades simultaneously [11]. Unlike many conventional cytotoxic agents that cause dose-limiting toxicities, such as myelosuppression, nausea, and organ damage, phytochemicals have been shown in preclinical and early clinical contexts to be well tolerated, often eliciting only mild adverse effects even at relatively high doses, which may support better patient tolerability and quality of life during extended treatment courses [12]. This low toxicity is further supported by epidemiological evidence linking diets rich in phytochemical-containing foods to reduced cancer incidence and limited adverse outcomes, as well as by reports that phytochemicals do not significantly damage normal tissues in vivo [13].

Multiple preclinical studies have shown that dietary phytochemicals can directly impact key CSC features, such as self-renewal, stemness signaling, migration, invasion, and chemoresistance, by modulating Wnt/β-catenin, Notch, Hedgehog, STAT3, and other pathways that underlie CSC survival and therapy escape [14]. These features make them particularly attractive in the context of CSCs, where redundancy and crosstalk among pathways present major therapeutic challenges. Indeed, dietary and medicinal phytochemicals have shown promising effects in preclinical models of CSC suppression by modulating self-renewal, inducing differentiation or apoptosis, reversing drug resistance, and disrupting niche signals [15,16]. In addition, there is emerging evidence that certain phytochemicals can mitigate the toxic side effects associated with chemotherapy and radiotherapy through antioxidant, anti-inflammatory, and immunomodulatory mechanisms, thereby potentially enhancing overall treatment tolerability [17].

Several prominent phytochemicals, often referred to as the “big five”, have been repeatedly studied for their anti-CSC activities: curcumin, epigallocatechin-3-gallate (EGCG), sulforaphane, resveratrol, and genistein. These compounds have been shown to interfere with critical CSC pathways. For instance, curcumin and sulforaphane suppress Wnt/β-catenin signaling; resveratrol and genistein modulate Notch and Hedgehog signaling; EGCG impacts PI3K/Akt and STAT3 axes [18]. Preclinical models, including sphere formation assays, ALDH^+^ or CD44^+^ CSC fractions, and in vivo tumorigenicity assays, provide compelling evidence that these phytochemicals reduce CSC populations and sensitize them to standard chemotherapy [4,19].

Despite their promise, the translation of phytochemicals into clinical anti-CSC agents remains challenging. Key obstacles include poor bioavailability, rapid metabolism, suboptimal pharmacokinetics, and variability in botanical preparations [15]. Clinical translation is also limited by the need for rigorous proof that phytochemicals not only suppress CSC markers but actually improve treatment outcomes and prevent relapse in patients, an area where current evidence remains preliminary [18]. Moreover, the complexity of CSC signaling, with extensive crosstalk and compensatory feedback among pathways, requires more precise mechanistic understanding and rational combinations to avoid unintended resistance [6,7]. In response to these challenges, recent research is pushing two converging fronts: (i) in-depth molecular elucidation of how phytochemicals engage CSC-specific signaling networks (e.g., identifying upstream transcription factors, miRNAs, or long non-coding RNAs that mediate phytochemical effects), and (ii) advanced delivery strategies and combinatorial formulations (e.g., nanocarriers, co-delivery with chemotherapeutics) to enhance the stability, bioavailability, and targeting of these compounds. For example, a 2025 review in Discover Oncology highlighted the capacity of curcumin, resveratrol, EGCG, and sulforaphane to inhibit CSC proliferation, modulate the microenvironment, and reverse drug resistance, but also stressed the need for innovative drug delivery platforms [15]. Given this evolving landscape, a comprehensive synthesis of current evidence is both timely and necessary.

In this review, we systematically examine recent high-impact studies on phytochemical-mediated targeting of CSCs, delineate the molecular mechanisms involved, assess functional assays used to evaluate anti-CSC activity, and propose translational strategies as well as future research directions. By explicitly emphasizing the central role of CSCs in treatment failure and recurrence, this work underscores the therapeutic imperative of targeting these cells alongside tumor bulk to achieve durable cancer remission. By doing so, we aim to provide a rigorous resource for researchers and clinicians interested in harnessing plant-derived compounds to eliminate the root of cancer persistence. The literature discussed in this review predominantly encompasses studies published between 2018 and 2025, with an emphasis on recent high-impact research elucidating phytochemical-based strategies for targeting CSCs.

## 2. Characteristics of Cancer Stem Cells

**Figure 1:**
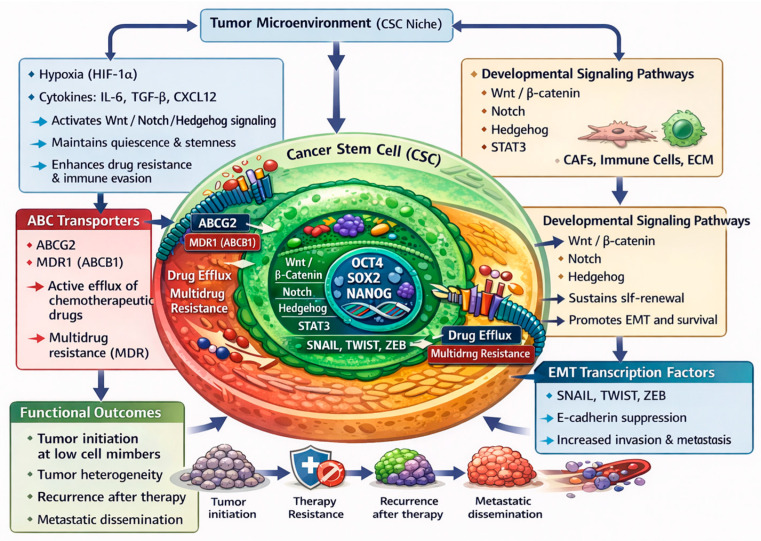
Flowchart representation of the defining characteristics and resistance mechanisms of cancer stem cells (CSCs). The schematic flowchart illustrates how signals from the tumor microenvironment (CSC niche) regulate CSC maintenance and therapeutic resistance. Hypoxic conditions (via HIF-1α), cytokines (IL-6, TGF-β, CXCL12), and stromal components such as cancer-associated fibroblasts (CAFs), immune cells, and extracellular matrix activate key developmental pathways including Wnt/β-catenin, Notch, Hedgehog, and STAT3. These pathways converge within CSCs to sustain self-renewal and stemness through nuclear transcription factors OCT4, SOX2, and NANOG. At the plasma membrane, overexpression of ATP-binding cassette (ABC) transporters (ABCG2 and MDR1/ABCB1) mediates active drug efflux, resulting in multidrug resistance. Concurrently, activation of EMT-associated transcription factors (SNAIL, TWIST, ZEB) suppresses E-cadherin, enhancing cellular plasticity, invasion, and metastatic potential. Collectively, these interconnected mechanisms promote high tumor-initiating capacity, therapy resistance, tumor heterogeneity, recurrence after treatment, and metastatic dissemination, underscoring CSCs as central drivers of cancer persistence and progression.

CSCs represent a highly specialized and resilient subpopulation within tumors, endowed with a constellation of biological traits that underlie their central role in malignancy, recurrence, and metastasis. One of their defining features is self-renewal, which is orchestrated by aberrant activation of developmental signaling pathways including Wnt/β-catenin, Notch, Hedgehog, and STAT3. These pathways maintain expression of core stemness transcription factors, such as OCT4, SOX2, and NANOG, enabling CSCs to perpetuate themselves over long periods [20,21,22]. Beyond self-renewal show in Figure 1, CSCs exhibit an exceptionally high tumor-initiating capacity even when present in very low numbers. In experimental models, a small fraction of CSCs can regenerate a heterogeneous tumor mass, mirroring the behavior of stem cells in normal development and underscoring their potency in driving tumorigenesis [23]. A major challenge in cancer therapy is posed by the drug resistance of CSCs, which arises in part from the overexpression of ATP-binding cassette (ABC) transporters such as ABCG2 and MDR1 (ABCB1). These efflux pumps actively eject chemotherapeutic agents, thereby reducing intracellular drug accumulation and promoting multidrug resistance (MDR) [15,24,25]. Complementing this defense, CSCs have a superior capacity for DNA repair, which enables them to survive genotoxic insults such as chemotherapy and radiotherapy. Enhanced DNA damage response pathways, checkpoint kinases, homologous recombination, and other repair machineries, are often upregulated in CSCs, aiding in their survival under therapeutic stress [26,27].

In addition, CSCs frequently adopt an epithelial–mesenchymal transition (EMT) phenotype, a plastic state characterized by increased motility and invasiveness. EMT not only promotes metastasis but also contributes to treatment resistance; transcription factors driven by Hedgehog, Notch, and Wnt signaling (such as SNAIL, TWIST, and ZEB) suppress E-cadherin, remodel cell adhesion, and foster a more migratory, stem-like behavior [21,22,25]. Another critical characteristic of CSCs is their dependence on a specialized niche. This microenvironmental niche, composed of stromal cells (e.g., cancer-associated fibroblasts), immune cells, extracellular matrix components, cytokines, and hypoxic zones, provides essential support for CSC maintenance. Under low oxygen, hypoxia-inducible factors like HIF-1α reinforce stemness signaling (Wnt, Notch, Hedgehog), promote quiescence, and boost drug resistance by reducing reactive oxygen species (ROS) levels [20,24,26]. Cytokines and growth factors secreted within the niche (such as IL-6, TGF-β, CXCL12) further sustain CSC self-renewal and survival, contributing to therapeutic resilience and immune evasion [20,23,28]. Collectively, these hallmarks, self-renewal via core signaling pathways, robust tumor initiation at low cell numbers, drug efflux-mediated resistance, elevated DNA repair, EMT-driven plasticity, and niche dependency, endow CSCs with a formidable capacity to survive conventional treatments, drive recurrence, and seed metastasis. Because these traits lie at the root of tumor persistence and spread, targeting CSCs has become a cornerstone of next-generation cancer therapy, motivating intensive research into modulators (such as phytochemicals) that can selectively disrupt these defining features.

## 3. Major Signaling Pathways Regulating CSC Biology

The Wnt/β-catenin pathway is perhaps the most extensively studied in this context. Aberrant activation of canonical Wnt signaling promotes β-catenin stabilization, nuclear translocation, and transcription of stemness-related genes (e.g., c-Myc, Cyclin D1), which underlies the capacity of CSCs to self-renew and resist differentiation [29,30,31]. Recent reviews and experimental studies in colorectal cancer (CC), breast cancer (BC), and prostate cancer (PC) models show that phytochemicals such as genistein, resveratrol, apigenin, and catechins can suppress β-catenin nuclear localization, downregulate Wnt target genes, and reduce sphere formation and tumor-initiating capacity [29,32,33]. Moreover, Wnt signaling’s crosstalk with other pathways, for example, through GSK-3β modulation by Akt, makes its inhibition especially attractive: in breast cancer, Akt-mediated phosphorylation of GSK-3β both activates β-catenin and amplifies Wnt signaling, and several phytochemicals can block this axis, thereby collapsing a key survival loop [34]. Notch signaling also plays a critical role in maintaining CSC populations by preventing differentiation and promoting survival. Ligand binding (Jagged, DLL) activates the γ-secretase–mediated release of the Notch intracellular domain (NICD), which translocates to the nucleus and drives transcription of stemness-associated genes such as Hes1 [31,33,35]. In high-impact BC models, phytochemicals like curcumin and its analogues have been shown to suppress Notch1 expression, reduce NICD levels, and limit mammosphere formation [31]. The ability of these compounds to inhibit γ-secretase–mediated cleavage suggests a direct interference with the canonical Notch activation machinery [35]. The Hedgehog (HH) pathway contributes to CSC maintenance via its classical effectors, Sonic Hedgehog (SHH), Patched (PTCH), Smoothened (SMO), and Gli transcription factors. Dysregulated HH signaling leads to GLI-mediated transcription of genes (such as Bcl-2, Cyclin D) that drive proliferation and self-renewal [29,32]. Phytochemicals have shown efficacy in disrupting this cascade. For example, studies in glioma and colorectal cancer models highlight that compounds like cyclopamine, curcumin, and resveratrol can inhibit SMO or GLI activity, thereby suppressing CSC-like traits, reducing sphere formation, and sensitizing cells to conventional therapies [33]. Indeed, a recent update in glioma therapy noted that such phytochemical modulation of Hedgehog signaling might counteract radio- and chemo-resistance [32].

**Figure 2:**
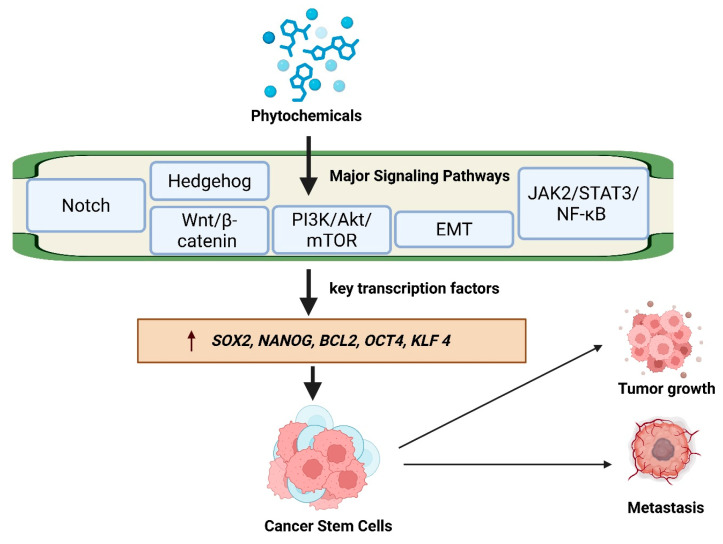
Activation of key signaling pathways drives transcriptional programs that sustain and expand CSC populations within tumors.

In parallel, the PI3K/Akt/mTOR pathway, a canonical survival and growth regulator, is hyperactivated in CSCs, often via loss of PTEN or upstream receptor-tyrosine-kinase stimulation [29]. Activation of Akt not only supports cell survival and proliferation but also feeds into Wnt signaling by phosphorylating/inhibiting GSK-3β, thus stabilizing β-catenin [34]. Phytochemicals such as piperlongumine and caffeic acid have been reported to inhibit PI3K/Akt/mTOR signaling in CSC-enriched populations, leading to reduced clonogenicity, induction of apoptosis, and diminished resistance [19,35]. Additionally, some studies show that compounds like plumbagin can upregulate PTEN, thereby restoring its negative control over Akt in PTEN-deficient cancer stem-like cells [32]. Finally, the JAK/STAT pathway, particularly via STAT3, is increasingly recognized for its role in CSC survival, inflammation-driven stemness, and drug resistance. Inflammatory cytokines such as IL-6 can activate JAK2/STAT3, promoting expression of Nanog, Sox2, and other stemness regulators, thereby reinforcing self-renewal and chemoresistance [36]. Phytochemicals including curcumin, morin, and ursolic acid have been shown to inhibit STAT3 phosphorylation, attenuate downstream target gene expression, and reduce CSC viability and tumorigenicity in preclinical models [19]. Importantly, these pathways do not operate in isolation: there is extensive crosstalk among Wnt, Notch, HH, PI3K/Akt, and STAT3 networks, which allows CSCs to bypass blockade of a single axis. For instance, Notch activation enhances Wnt signaling in some contexts, while PI3K/Akt fosters β-catenin stability, and STAT3 can cooperate with GLI transcription factors to maintain stemness [29,33,35]. Phytochemicals, by virtue of their multi-target modes of action, are uniquely suited to disrupt this integrated network: several compounds simultaneously modulate more than one pathway, preventing compensatory feedback and thereby reducing CSC fitness more effectively than agents that act on a single target [19]. In summary, recent high-impact studies underscore that phytochemicals target CSC biology most effectively by concurrently disrupting multiple core oncogenic pathways, especially Wnt/β-catenin, Notch, Hedgehog, PI3K/Akt/mTOR, and JAK/STAT show in Figure 2. This multi-pathway interference undermines the robustness and resilience of CSCs, suppressing their self-renewal, survival, and therapy resistance. Harnessing this broad-spectrum mechanism could make phytochemical-based interventions particularly powerful in eradicating CSC populations and improving long-term therapeutic outcomes.

### 3.1. Wnt/β-Catenin Pathway

The canonical Wnt/β-catenin signaling axis plays a central role in governing self-renewal, proliferation, and epithelial–mesenchymal transition (EMT) in cancer stem cells (CSCs). Under normal physiological conditions, Wnt ligands engage Frizzled receptors and LRP5/6 co-receptors, leading to inhibition of the β-catenin destruction complex and stabilization of cytoplasmic β-catenin. This stabilized β-catenin translocates to the nucleus and co-activates TCF/LEF transcription factors, driving expression of genes that promote cell-cycle progression (e.g., cyclin D1), survival (c-Myc), and stemness. In CSCs derived from BC, CC, and hepatocellular carcinoma (HCC), aberrant activation of the Wnt/β-catenin pathway has been widely documented. For instance, in colorectal cancer, hyperactivation of Wnt signaling is nearly ubiquitous and correlates with enhanced CSC propagation, EMT, chemoresistance, and metastasis [37,38]. In HCC, recent transcriptomic and genomic studies identify subtypes characterized by elevated β-catenin activity; nuclear translocation of β-catenin in these tumors supports proliferation and survival of HCC stem-like cells [39,40]. In breast CSCs, high expression of Wnt pathway components such as LEF1, TCF-4, and β-catenin has been correlated with stemness markers (e.g., CD44, ALDH1), and experimental suppression of Wnt ligands reduces both self-renewal capacity and tumorigenicity [41].

The Wnt/β-catenin pathway also drives EMT, a process by which CSCs acquire invasive and metastatic traits. Nuclear β-catenin induces EMT-related transcriptional programs that upregulate mesenchymal markers and repress epithelial ones, thereby enhancing plasticity and dissemination [42,43]. Through EMT, CSCs gain not only migratory capacity but also increased resistance to cytotoxic treatment, reinforcing their role in tumor relapse and metastasis. Phytochemicals have shown promising ability to target this pathway in CSCs. For example, in colorectal CSCs, proanthocyanidins derived from plants reduce β-catenin levels, suppress phosphorylated GSK-3β, and impair tumorsphere-forming ability [44]. In breast CSCs, alpha-hederin has been identified (via in silico docking and in vitro validation) as a potent Wnt/β-catenin inhibitor: it downregulates TCF/LEF target genes such as cyclin D1 and CD44, upregulates p53, and induces apoptosis in CSC-enriched populations [45]. In HCC, curcumin has been shown to interfere with Wnt signaling by reducing β-catenin accumulation, decreasing downstream targets such as c-Myc and cyclin D1, and suppressing migration, thereby restraining both proliferative and EMT capacities of CSC-like cells [46]. Taken together, these findings underscore that overactivation of the Wnt/β-catenin pathway is a hallmark of CSCs in breast, colorectal, and liver cancers, driving self-renewal, proliferation, and EMT. Importantly, phytochemicals can effectively disrupt this pathway at multiple levels, from ligand-receptor interactions to nuclear β-catenin function, making them compelling agents for CSC-targeted therapy.

### 3.2. Notch Pathway

The Notch signaling cascade is a conserved cell–cell communication system that critically controls cell fate decisions, lineage commitment, and stemness programs, and its aberrant activation is now recognized as a central driver of aggressive cancer phenotypes. In canonical Notch signaling, ligand engagement (Jagged, DLL) triggers proteolytic cleavages culminating in release of the Notch intracellular domain (NICD), which translocates to the nucleus to assemble a transcriptional complex that upregulates genes promoting self-renewal and suppressing differentiation. Persistent or ectopic Notch activation in tumors preserves an undifferentiated, stem-like state and is mechanistically linked to enhanced invasion, angiogenesis, and blockade of terminal differentiation in multiple malignancies [47,48]. Functionally, Notch sustains CSC populations by maintaining expression of stemness transcription factors and by repressing differentiation programs; this enables tumor cells to retain high tumor-initiating capacity, resist cytotoxic stress, and repopulate tumors after therapy. Importantly, Notch signaling also coordinates pro-invasive and pro-angiogenic programs: NICD-driven transcription induces EMT regulators and matrix-remodeling genes that facilitate invasion, and reciprocal interactions between Notch and VEGF signaling in the tumor endothelium regulate angiogenic sprouting and the formation of a vascular niche that supports CSC maintenance. These pleiotropic roles explain why elevated Notch activity correlates with poor prognosis, increased metastasis, and aggressive clinical behavior in several cancer types [47,49].

Recent mechanistic and translational studies further show that targeting Notch at multiple levels, ligand availability, γ-secretase–mediated NICD release, NICD stability, or the Notch transcriptional complex, reduces CSC frequency, impairs tumorsphere formation, and restores differentiation in preclinical models. Natural bioactive compounds (phytochemicals) such as curcumin, resveratrol, EGCG and related polyphenols have been reported to downregulate Notch receptors and ligands, inhibit γ-secretase activity or promote NICD degradation, thereby diminishing Notch transcriptional output and associated stemness phenotypes. These phytochemical interventions not only blunt self-renewal but also attenuate Notch-driven invasion and angiogenesis in vitro and in vivo, supporting their potential as multi-target agents to reprogram CSCs toward differentiation and vulnerability [50,51]. Because Notch exerts context-dependent roles (oncogenic in some tissues and tumor-suppressive in others), recent reviews emphasize the necessity of tissue- and tumor-specific mechanistic profiling before therapeutic targeting; nonetheless, the preponderance of evidence in breast, glioma, hepatocellular and several epithelial cancers positions Notch inhibition, including modulation by phytochemicals, as a promising strategy to dismantle CSC-driven invasion, angiogenesis, and differentiation blockade. Continued integration of pathway-level studies with careful pharmacologic and delivery optimization will be required to translate these findings safely and effectively [47,52].

### 3.3. Hedgehog (Hh) Pathway

The Hh signaling pathway has emerged as a pivotal regulator of CSC biology, critically orchestrating self-renewal, survival, and tumorigenic potential in multiple malignancies. In PC, for instance, recent studies highlight a finely tuned miRNA–Hh regulatory axis: miR-630 suppresses CSC properties by targeting PRKCI, which in turn attenuates Hh-GLI signaling. Loss of miR-630 leads to enhanced GLI1 activity, promoting stemness and tumor-initiating capacity in pancreatic CSCs, thereby underscoring that GLI-driven Hh output is necessary for their renewal [53]. Moreover, phytochemical intervention further supports the dependence of pancreatic CSCs on this pathway: treatment with sulforaphane (SFN) has been shown to inhibit SHH-GLI signaling in pancreatic tumor spheres, leading to downregulation of stemness factors (e.g., Nanog, Oct-4) and induction of apoptosis, demonstrating that active Hh signaling is required for CSC maintenance [54]. In OC, particularly high-grade serous ovarian carcinoma (HGSOC), aberrant Hh signaling is likewise central to CSC enrichment. Pharmacological inhibitors of Hh (such as GANT61, LDE225, and vismodegib) markedly reduce spheroid formation and diminish the ALDH1A1-positive CSC fraction in primary malignant ascites-derived cells [55]. Under hypoxic conditions, a common microenvironmental stress in ovarian tumors, Hh activity synergizes with PI3K/AKT signaling to support vasculogenic mimicry (VM) in CSCs; co-inhibition of PI3K and Hh under hypoxia suppresses VM, proliferation, and invasiveness of ovarian stem-like cells, strongly suggesting that Hh signaling is functionally required for CSC-driven plasticity [56].

In the context of BCs, particularly glioblastoma (GBM), the role of Hh signaling in glioma stem cells (GSCs) is increasingly well characterized. The transcription factor ISL1 has been identified as a critical upstream activator of the SHH/GLI1 axis in patient-derived GSCs: ISL1 knockdown significantly reduces GLI1 expression, diminishes cell viability and proliferation, and increases apoptosis, while addition of recombinant SHH partially rescues these effects [57]. Further, epigenetic regulation tightly modulates GLI1 expression in GSCs: simultaneous inhibition of BRD4 (by JQ1) and HDAC3 markedly suppresses GLI1 transcription, disrupts the GLI1–IL6–STAT3 signaling loop, and synergistically abrogates GSC growth in vitro and in vivo [58]. These data reinforce that not only canonical Hh activity but also chromatin-level control of GLI1 is indispensable for GSC self-renewal. Beyond intrinsic regulation, the microenvironment also modulates Hh-dependent CSC behavior in the brain. Co-culture of GSCs with astrocyte organoids reveals that SHH secreted by tumor cells stimulates astrocytes to produce morphogens, which in turn restrain GSC proliferation. Paradoxically, pharmacological blockade of SMO via vismodegib enhances GSC proliferation in this context, suggesting a complicated paracrine feedback mechanism in which ASTROCYTES relay Hh signals to constrain stem-like cell overgrowth [59]. Taken together, these lines of evidence from pancreatic, ovarian, and brain tumors converge on a compelling paradigm: Hh signaling, particularly via GLI1, is not merely a bystander but a core determinant of CSC fate and function. Its activity is tightly regulated by microRNAs, epigenetic modulators, and niche interactions, all of which integrate to sustain CSC renewal, plasticity, and resilience in tumor contexts. These multifaceted controls of Hh-GLI signaling make it an especially promising and nuanced target for therapies aimed at eliminating CSCs and preventing tumor relapse.

### 3.4. PI3K/Akt/mTOR Pathway

The PI3K–Akt–mTOR (PAM) signaling axis functions as a master regulator that sustains CSC survival, drives metabolic reprogramming, and enforces resistance to apoptosis across tumor types, thereby underpinning key facets of CSC-mediated therapy failure and relapse. Activation of PAM signaling in CSCs promotes prosurvival programs by phosphorylating downstream effectors that inhibit pro-apoptotic machinery and favor cell-cycle progression; consequently, CSC populations isolated from patient tumors and model systems exhibit elevated PI3K/Akt activity and mTORC1/2 signaling relative to bulk tumor cells, and this elevation correlates with enhanced clonogenicity and decreased sensitivity to cytotoxic agents [60,61]. Beyond classical survival signaling, the PAM axis is a central driver of metabolic plasticity in CSCs. By stimulating glucose uptake and glycolytic flux, up-regulating lipid biosynthesis, and coordinating mitochondrial function through mTOR-dependent control of anabolic processes, PI3K/Akt/mTOR enables CSCs to rewire metabolism toward biosynthesis and redox balance required for long-term self-renewal under nutrient or oxygen stress. Such metabolic reprogramming is a reproducible feature in gliomas and other aggressive tumors, where PAM activation sustains alternative metabolic routes (e.g., pentose phosphate pathway, fatty-acid utilization) that support CSC proliferation and survival in hostile microenvironments [62,63].

Functionally, this coupling of survival and metabolism translates into pronounced resistance to apoptosis and conventional therapies. In ovarian cancer, for example, hyperactivation of PI3K/Akt/mTOR in the CSC fraction is associated with chemoresistance, epithelial–mesenchymal transition (EMT), and maintenance of stem markers (such as ALDH1A1), and pharmacologic or genetic inhibition of PAM signaling reduces CSC frequency, reverses EMT phenotypes, and restores chemosensitivity in preclinical models. Similar therapeutic vulnerabilities have been reported in multiple other malignancies: inhibition of PI3K/Akt or mTOR complexes selectively compromises CSC viability, lowers clonogenic output, and synergizes with cytotoxic agents to increase apoptosis, demonstrating that PAM signaling is both a mediator of therapy resistance and a tractable target to disable CSC survival [64,65]. Importantly, PAM activity in CSCs is embedded within a dense network of cross-talks, integrating signals from receptor tyrosine kinases, nutrient sensors, hypoxia-induced HIF-1α, and other stemness pathways, so that single-node inhibition can be circumvented by compensatory feedbacks. Consequently, recent translational work emphasizes combined targeting strategies (for example, dual PI3K/mTOR inhibitors or PAM blockade paired with metabolic or epigenetic modulators) to collapse the metabolic and anti-apoptotic scaffolds that CSCs depend upon. These multi-modal approaches have produced the most consistent suppression of CSC self-renewal and survival in contemporary preclinical studies, arguing that effective eradication of CSCs will require therapies that simultaneously disable PAM-driven prosurvival signals and the metabolic programs they sustain [60,66].

### 3.5. STAT3/NF-κB Pathways

Chronic inflammation fosters a tumor-permissive milieu by co-opting two master transcriptional regulators, STAT3 and NF-κB, that together drive an inflammation-to-stemness program essential for malignant progression. Persistent activation of STAT3 in tumor cells and stromal/immune compartments promotes transcription of genes that sustain proliferation, survival, and pluripotency (e.g., SOX2, NANOG, BCL2), while NF-κB amplifies a pro-inflammatory secretome (IL-6, IL-8, TNFα) that reinforces STAT3 signaling in an autocrine/paracrine loop. This reciprocal reinforcement establishes a feed-forward circuit that enriches and maintains CSC pools, conferring resistance to apoptosis and standard therapies and driving metastatic competence. Contemporary mechanistic and translational studies underline that breaking this STAT3–NF-κB alliance is central to dismantling inflammation-driven CSC programs [67,68]. In PC, where chronic pancreatitis and desmoplastic stroma are frequent, STAT3 activation is tightly linked to CSC phenotypes and therapy resistance. Recent reviews and experimental studies report that inhibition of STAT3 signaling reduces sphere-forming ability, diminishes ALDH^high/CD44^+^ CSC fractions, and restores sensitivity to chemotherapeutics in PDAC models, while stromal and immune-derived cytokines maintain STAT3 phosphorylation in CSCs. These data indicate that STAT3 is both necessary for intrinsic CSC survival programs and a mediator of microenvironmental signals that sustain pancreatic CSC renewal [69,70]. For OC, multiple recent investigations demonstrate that NF-κB and STAT3 cooperate to sustain stem-like properties in spheroid cultures and patient-derived ascites. Inhibition of NF-κB signaling or genetic disruption of components that link NF-κB to chromatin regulators (for example HOTAIR–EZH2 axes that engage NF-κB/ALDH1A1 programs) substantially reduces CSC markers, clonogenicity, and invasive behavior. Under hypoxia or post-chemotherapy conditions, both of which are common in the ovarian tumor microenvironment, activation of NF-κB/STAT3 signaling promotes vasculogenic mimicry and EMT-associated plasticity, linking inflammation directly to the emergence and maintenance of therapy-resistant CSCs [64,71].

In brain tumors, emerging work has clarified context-specific STAT3 roles in glioma stem cell (GSC) biology. Mechanistic studies identify upstream regulators (e.g., integrin-linked kinase, ILK) that activate STAT3 to control GSC plasticity and lineage transitions; STAT3 inhibition reduces self-renewal, promotes apoptotic responses in GSCs, and attenuates tumorigenic growth in orthotopic models. NF-κB intersects with STAT3 in the neuroinflammatory niche to shape cytokine networks that preserve GSC pools and protect them from therapy-induced cell death, highlighting the dual requirement for both pathways in sustaining aggressive brain CSC phenotypes [72,73]. Therapeutically, the STAT3–NF-κB axis offers multiple vulnerable nodes. Selective STAT3 inhibitors and approaches that disrupt NF-κB-driven cytokine loops show promise preclinically, but single-agent strategies are often blunted by compensatory signaling; consequently, combination regimens that co-target inflammatory signaling, epigenetic regulators, or metabolic dependencies produce the most robust depletion of CSCs in recent studies. Importantly for this review, several phytochemicals and natural compounds (for example EGCG and related polyphenols) have been reported to attenuate STAT3/NF-κB signaling and reduce CSC markers and sphere formation in preclinical models, suggesting a feasible translational route to modulate inflammation-driven stemness with lower-toxicity agents. Collectively, the literature positions STAT3 and NF-κB as central, cooperating drivers of inflammation-driven CSC biology and as high-value targets for interventions designed to prevent recurrence and metastasis [16,74].

### 3.6. EMT-Associated Pathways

EMT and its core transcriptional effectors, TGF-β, Snail, Twist and ZEB1, constitute a conserved program that confers mesenchymal traits and high plasticity to tumor cells, thereby enabling the acquisition and maintenance of CSC properties. TGF-β acts as a master upstream inducer: through canonical SMAD signaling and extensive cross-talk with non-SMAD pathways, TGF-β drives transcriptional reprogramming that up-regulates EMT transcription factors and suppresses epithelial markers, creating a permissive state for stemness, invasion and immune evasion in diverse cancers. Recent mechanistic and in vivo studies demonstrate that TGF-β–SMAD signaling not only initiates phenotypic conversion toward a mesenchymal program but also stabilizes that state in the presence of microenvironmental stressors, linking chronic stromal cues to durable CSC enrichment [75,76]. Downstream EMT transcription factors execute and refine this plasticity. Snail family proteins repress epithelial junctional components and activate stemness-associated gene networks, while Twist promotes mesenchymal identity and motility and, in multiple model systems, is required for dissemination and metastatic competence; genetic ablation of Twist in tumor cells markedly reduces plasticity and metastatic seeding, underscoring its functional importance for CSC-like behavior in vivo. These factors do not act in isolation but cooperate: Snail and Twist can converge on regulatory elements that induce ZEB1 expression, producing a layered transcriptional architecture that locks cells into a mesenchymal, stem-prone configuration [77,78].

**Figure 3:**
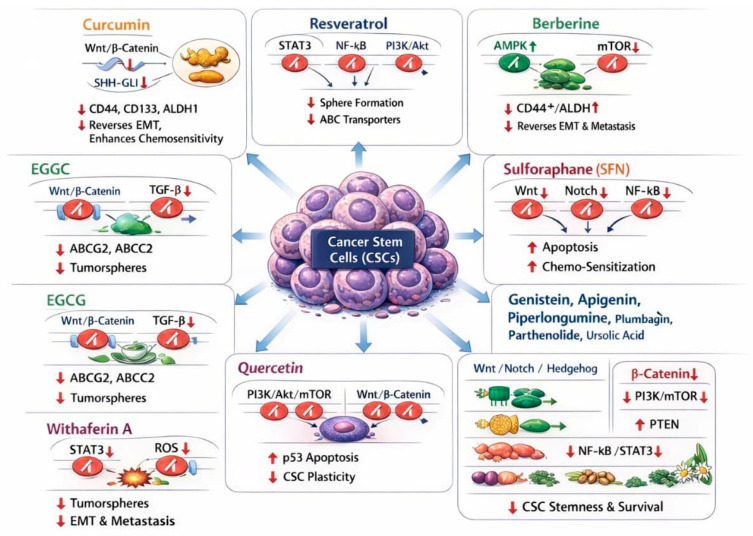
Schematic representation of the molecular pathways targeted by major phytochemicals in CSCs. The central panel represents CSCs, while surrounding panels illustrate the specific signaling pathways modulated by individual phytochemicals. Curcumin suppresses Wnt/β-catenin and Hedgehog (SHH–GLI) signaling, leading to reduced CSC markers (CD44, CD133, ALDH1), reversal of EMT, and enhanced chemosensitivity. Resveratrol inhibits STAT3, NF-κB, and PI3K/Akt pathways, thereby suppressing tumorsphere formation and ABC transporter–mediated drug resistance. Berberine activates AMPK and inhibits mTOR signaling, resulting in decreased CD44^+^/ALDH^high CSC populations and inhibition of EMT and metastasis. EGCG suppresses Wnt/β-catenin and TGF-β–induced EMT and inhibits ABC transporters (ABCG2, ABCC2), reducing tumorsphere formation. Sulforaphane (SFN) inhibits Wnt/β-catenin, Notch, and NF-κB pathways, induces apoptosis in CSCs, and sensitizes tumor cells to chemotherapeutic agents. Withaferin A inhibits STAT3 signaling and induces ROS-mediated apoptosis, leading to reduced tumorsphere formation, EMT, and metastatic potential. Quercetin inhibits PI3K/Akt/mTOR and Wnt/β-catenin pathways and promotes p53-mediated apoptosis, reducing CSC plasticity. Other phytochemicals, including genistein, apigenin, catechins, piperlongumine, caffeic acid, plumbagin, parthenolide, and ursolic acid, collectively target Wnt, Notch, Hedgehog, PI3K/Akt/mTOR, NF-κB, and STAT3 signaling, thereby suppressing CSC stemness, survival, and therapeutic resistance. The regulatory effects in the figure are conveyed exclusively by arrow direction and symbols, where downward arrows (↓) indicate inhibition and upward arrows (↑) indicate activation.

ZEB1 occupies a central nodal position linking EMT to therapy resistance and metabolic reprogramming in CSCs. High-resolution functional studies have shown that ZEB1 not only represses epithelial differentiation programs but also reorganizes lipid metabolism and redox balance in mesenchymal cells, creating vulnerabilities (for example altered ferroptosis sensitivity) that are intimately tied to the EMT state. This work highlights that ZEB1-driven EMT is a multifaceted program: it enforces stemness and invasiveness while simultaneously remodeling cellular metabolism to support long-term survival in hostile microenvironments [79]. Clinically and translationally relevant is the observation that EMT-associated programs confer therapeutic refractoriness by several mechanisms: promotion of quiescence, up-regulation of drug-efflux and anti-apoptotic pathways, and facilitation of niche interactions that shield CSCs from immune elimination and cytotoxic insults. Large-scale reviews and recent experimental reports emphasize that EMT–CSC coupling is a dynamic, reversible process, cells can oscillate between epithelial and mesenchymal states, so that interventions must address not only static EMT markers but the signaling circuits and microenvironmental inputs (TGF-β, hypoxia, inflammatory cytokines) that drive state transitions [80,81]. As shown in Figure 3, the molecular pathways targeted by major phytochemicals in CSCs are illustrated schematically.

## 4. Phytochemicals with Anti-CSC Activity

In addition to the widely studied “big five” phytochemicals, this review also includes plant-derived compounds, such as curcumin, EGCG, sulforaphane, resveratrol, withaferin A, berberine, and quercetin, because recent high-quality studies consistently demonstrate their relevance to CSC biology through distinct yet complementary mechanisms. These phytochemicals were selected not merely on the basis of tradition or prevalence, but because they consistently emerge in recent high-impact research as compounds with reproducible and mechanistically substantiated anti-CSC activity across diverse cancer models. These agents have each been shown to modulate multiple pathways that are central to CSC self-renewal, survival, therapy resistance, and metastatic capacity, including Wnt/β-catenin, Notch, Hedgehog, PI3K/Akt/mTOR, STAT3, and EMT-associated signaling networks, and they reduce CSC marker expression and functional phenotypes in both in vitro and in vivo contexts [4,19]. Curcumin and EGCG, for example, have been repeatedly documented to inhibit CSC proliferation and sphere formation and, in some cases, to enhance chemosensitivity, while sulforaphane selectively targets ALDH-positive CSC populations and disrupts key self-renewal circuits derived from cruciferous vegetable sources [14,82]. Resveratrol’s capacity to impair CSC survival and reduce metastatic traits via multiple signaling axes, and genistein’s documented inhibition of Hedgehog and STAT signaling in CSC contexts, further underscore their role as prototype phytochemicals with broad anti-CSC potential [14]. These “big five” compounds also feature prominently in systematic reviews and meta-analyses that collate both mechanistic and translational evidence, which distinguishes them from many other natural products that show only isolated effects or limited translational evaluation, and supports our emphasis on them as representative phytochemical candidates with the greatest evidentiary weight for CSC targeting [19,82].

### 4.1. Curcumin

Curcumin, the principal bioactive diarylheptanoid extracted from *Curcuma longa* (turmeric), has accumulated substantial preclinical evidence as a multi-modal inhibitor of CSC programs across several tumor types. Mechanistically, curcumin suppresses developmental signaling networks that sustain stemness, notably Wnt/β-catenin and Sonic Hedgehog (SHH–GLI) axes, producing reduced nuclear β-catenin and GLI1 expression, impaired tumoursphere formation and downregulation of downstream pluripotency targets. These pathway effects have been repeatedly documented in mechanistic and review studies, which show that curcumin’s inhibition of Wnt and Hh signaling underlies much of its capacity to decrease CSC self-renewal [83,84]. Consistent with pathway suppression, multiple experimental reports demonstrate that curcumin reduces canonical CSC markers and functional CSC readouts in diverse models. Treatment with curcumin or optimized curcumin analogues/nanopreparations lowers the proportions of CD44- and CD133-expressing cells and diminishes ALDH1 activity in breast, colorectal, pancreatic and glioma model systems, with attendant reductions in clonogenicity and in vivo tumor-initiating capacity in several studies. These marker changes are observed in both adherent cultures and patient-derived spheroids, indicating that curcumin targets intrinsic CSC traits rather than acting solely on bulk proliferating cells [85,86]. Curcumin also counteracts EMT, a program closely linked to acquisition of stemness. In vitro and in vivo studies show that curcumin downregulates EMT transcription factors such as Snail and Twist, restores epithelial markers, and reduces migratory/invasive phenotypes; in tumor models these molecular shifts coincide with a loss of sphere-forming ability and decreased metastatic behavior, indicating that EMT reversal is a mechanistic route by which curcumin diminishes CSC plasticity [87,88].

Importantly for translational intent, curcumin enhances the effectiveness of standard chemotherapeutics: preclinical combination studies report that curcumin potentiates cisplatin- and paclitaxel-induced apoptosis, lowers the effective doses required for tumor cell kill, and reduces the residual CSC fraction after chemotherapy. Mechanistic analyses attribute this chemosensitization to curcumin’s inhibition of survival signaling, suppression of drug-efflux mediators and depletion of the therapy-resistant CSC compartment, together producing more durable reductions in clonogenic regrowth in multiple cancer models [89,90]. Despite promising anti-CSC activity, curcumin’s clinical translation has been constrained by poor aqueous solubility and rapid metabolism. Recent advances in formulation science (liposomal, polymeric and other nanodelivery systems) have improved curcumin bioavailability and tumor delivery and frequently enhance its anti-CSC efficacy in preclinical studies, strengthening the rationale for continued development of curcumin-based combinations aimed at eradicating CSCs and preventing relapse. Taken together, the current literature supports curcumin as a prototypical phytochemical that inhibits Wnt/β-catenin and Hedgehog signaling, downregulates CD44/CD133/ALDH1, reverses EMT via suppression of Snail and Twist, and potentiates paclitaxel and cisplatin activity across breast, colon, pancreatic and glioma models, making it a compelling candidate for CSC-targeted combination strategies [90,91].

### 4.2. Resveratrol

Resveratrol, a stilbene polyphenol abundant in grapes and berries, exerts multifaceted anti-CSC activity by converging on inflammation- and survival-linked signaling networks that sustain stemness. Mechanistic investigations in recent years show that resveratrol suppresses STAT3 and NF-κB transcriptional activity and down-regulates PI3K/Akt signaling, thereby interrupting the cytokine-driven feed-forward loops and prosurvival programs that maintain CSC pools. Functionally, this signaling suppression is associated with decreased expression of stemness genes and diminished activation of prosurvival effectors, producing a biochemical environment hostile to long-term self-renewal and therapy resistance [92,93]. Consistent with pathway inhibition, multiple recent preclinical studies report that resveratrol markedly impairs tumorsphere/mammosphere formation and other CSC functional readouts across tumor types. In breast cancer models, resveratrol reduces mammosphere formation, induces autophagy in the CSC compartment, and lowers in vivo tumor-initiating potential; similar sphere-forming deficits and reductions in clonogenicity have been documented in prostate and colorectal cancer models, indicating that resveratrol interferes with the core self-renewal machinery across diverse CSC contexts. These effects frequently coincide with induction of autophagy or apoptosis in the residual CSC fraction and with suppression of Wnt/β-catenin and other stemness pathways that cross-talk with STAT3 and PI3K/Akt [94,95].

A second, clinically relevant axis of resveratrol’s anti-CSC action is its ability to down-modulate drug-efflux transporters and other mediators of multidrug resistance. Several mechanistic and review articles note that resveratrol and related natural compounds decrease expression or activity of ABC family transporters (including ABCB1/P-gp and ABCG2/BCRP) in chemotherapy-exposed colorectal and breast cancer models, contributing to restored intracellular drug accumulation and enhanced chemosensitivity of the previously drug-resistant CSC fraction. This transporter suppression, together with inhibition of PI3K/Akt (a known regulator of ABC transporter expression and activity), provides a rational explanation for observations that resveratrol diminishes residual CSCs after cytotoxic treatment [96,97]. Taken together, contemporary evidence positions resveratrol as a robust phytochemical antagonist of inflammation-linked and prosurvival signaling (STAT3, NF-κB, PI3K/Akt), an inhibitor of CSC self-renewal in sphere assays and xenografts, and a suppressor of ABC transporter–mediated drug efflux, with reproducible activity reported in breast, prostate and colorectal CSC models. Although bioavailability and pharmacokinetics remain translational challenges, the consistent mechanistic signatures across multiple recent studies support further development of optimized resveratrol formulations or combination regimens aimed at depleting CSCs and overcoming therapy resistance [92,93,94].

### 4.3. Berberine (BBR)

BBR, an isoquinoline alkaloid isolated from Berberis vulgaris and Coptis chinensis, has emerged as a promising phytochemical for targeting CSC programs by reprogramming tumor bioenergetics, suppressing stemness markers and impairing metastatic phenotypes. Mechanistically, a consistent theme across recent preclinical studies is that BBR perturbs the AMPK–mTOR signaling node: BBR promotes AMPK activation and consequent inhibition of mTORC1-driven anabolic signaling, a shift that favors autophagy, reduces protein synthesis and collapses the anabolic support that CSCs require for long-term self-renewal. This metabolic re-wiring both lowers CSC viability in vitro and sensitizes tumor-initiating cells to stress in vivo, and has been documented in hepatocellular carcinoma and other liver cancer models where BBR treatment decreased tumor growth and induced autophagy-associated tumor suppression [98,99]. Convergent functional evidence shows that BBR reduces canonical CSC markers and functional readouts across multiple tumor types. In recent breast cancer studies, BBR decreased the proportion of CD44^+^ and ALDH^high cells, suppressed mammosphere formation and attenuated tumor-initiating potential in xenograft assays, implicating a direct effect on the therapy-resistant CSC compartment. Comparable reductions in CD44/CD133 expression and ALDH activity have been observed in liver and ovarian model systems, where BBR treatment corresponded with diminished clonogenicity and impaired serial propagation, hallmarks of CSC depletion. These marker and functional changes have been reproduced using both free BBR and improved formulations (for example liposomal or nanoparticle preparations), which often enhance tumor delivery and anti-CSC potency [99,100].

Beyond marker reduction, BBR exerts clear anti-metastatic and EMT-reversing effects that are highly relevant to CSC biology. Multiple recent reports show that BBR suppresses EMT transcriptional programs (including reductions in Snail, Twist and vimentin expression), inhibits migration and invasion in vitro, and reduces metastasis in orthotopic and experimental metastasis models of ovarian and breast cancer. In ovarian cancer models where chemotherapy paradoxically induces GLI1-dependent EMT and CSC-like traits, BBR attenuated chemotherapy-exacerbated migration and reversed EMT features, effects tied to suppression of GLI1/BMI1 signaling and restoration of epithelial markers, thereby demonstrating that BBR can both prevent therapy-induced CSC enrichment and directly inhibit metastatic competency [101,102]. Mechanistic breadth is an asset: in addition to the AMPK–mTOR axis, BBR influences inflammatory and epigenetic regulators that intersect with stemness circuits. Reviews and mechanistic studies report modulation of apoptosis regulators, oxidative stress pathways and microRNA networks following BBR exposure, all of which contribute to the observed reductions in CSC fractions and tumor-initiating ability across liver, breast and ovarian systems. Importantly, several studies highlight that optimized BBR delivery (nano-formulations, liposomal encapsulation) amplifies its anti-CSC effects and mitigates pharmacokinetic limitations that have historically constrained clinical translation [99,103]. Taken together, recent literature positions berberine as a multifaceted inhibitor of CSC biology: bsy activating AMPK and suppressing mTORC1 activity, downregulating CD44^+^/ALDH^high CSC populations, reversing EMT programs and reducing metastatic spread, BBR shows consistent, cross-tumor evidence of CSC suppression in liver, breast and ovarian models. These convergent preclinical data justify further development of BBR, particularly in improved formulations and in combination with standard therapies, to exploit its capacity to dismantle the metabolic and phenotypic scaffolds that sustain therapy-resistant CSCs [98,100,101].

### 4.4. Epigallocatechin-3-Gallate (EGCG)

EGCG, the major catechin of green tea (*Camellia sinensis*), shows consistent and reproducible anti-CSC activity across lung, breast and colorectal cancer models through coordinated modulation of developmental signaling, drug-resistance machinery and self-renewal programs. Mechanistic studies and recent reviews converge on two principal axes of EGCG action relevant to CSC biology: inhibition of Wnt/β-catenin signaling and interruption of TGF-β–driven EMT/stemness programs. In colorectal cancer models, EGCG reduced nuclear β-catenin levels, downregulated downstream Wnt targets and markedly impaired spheroid formation and expression of CSC markers (for example, CD44, LGR5 and OCT4), establishing Wnt inhibition as a major mechanism by which EGCG depletes colorectal CSCs [104,105]. Parallel work in lung and breast systems corroborates these pathway effects and demonstrates potent functional consequences for self-renewal. Enriched lung cancer stem-like cells treated with EGCG formed significantly smaller and fewer tumorospheres, showed lower proliferation and increased apoptosis, and displayed downregulation of CLOCK and other stemness-associated transcripts in one recent study, findings that link EGCG’s molecular impact to loss of tumor-initiating capacity in non-small cell lung cancer. Likewise, comparative analyses of catechins identified EGCG as the most effective inhibitor of sphere formation in NSCLC and breast CSC assays, and several reports document that EGCG attenuates TGF-β signaling and EMT marker expression (for example, reduced vimentin and Snail), thereby reversing the mesenchymal phenotypes that underpin CSC plasticity and dissemination [106,107].

A clinically relevant facet of EGCG’s anti-CSC profile is its ability to antagonize ABC transporter–mediated drug efflux, a key mechanism of chemoresistance in CSCs. Preclinical studies and reviews report that EGCG decreases the expression and/or activity of ABC family transporters (including ABCG2 and ABCC2) in multiple tumor models, improving intracellular accumulation of chemotherapeutics and sensitizing CSC-enriched populations to standard agents. This transporter suppression, when combined with EGCG’s inhibition of survival pathways (STAT3/NF-κB and PI3K/Akt in some contexts), provides a mechanistic basis for the repeatedly observed reductions in residual CSC fractions after chemotherapy in co-treatment studies [108,109]. Taken together, a body of complementary studies, spanning mechanistic interrogations, spheroid and side-population assays, and xenograft validations, supports EGCG as a multifaceted inhibitor of CSCs in lung cancers (LCs), BCs and CCs. Its primary molecular actions (suppression of Wnt/β-catenin and TGF-β/EMT signaling), consistent reduction in tumorsphere formation, and down-modulation of ABC transporters make EGCG a compelling candidate for incorporation into CSC-targeted combination regimens, particularly where formulation strategies (nanodelivery, prodrugs) can overcome its pharmacokinetic limitations.

### 4.5. Sulforaphane (SFN)

SFN, an isothiocyanate enriched in broccoli sprouts, has emerged as one of the most compelling phytochemicals for selectively targeting CSCs. Across multiple tumor models SFN exerts a coordinated blockade of developmental and inflammatory stemness circuits, notably Wnt/β-catenin, NF-κB and Notch signaling, producing reproducible loss of self-renewal, induction of apoptosis in CSC populations, and diminished tumor-initiating capacity. In colorectal systems SFN has been shown to inhibit Wnt/β-catenin transcriptional activity downstream of β-catenin stabilization, reducing tumoursphere formation and expression of LGR5 and other intestinal CSC markers [110]. Functionally, SFN consistently reduces CSC functional readouts (sphere formation, side-population frequency, and in vivo tumor initiation) and drives apoptotic programs in the residual stem-like cells. Studies in breast cancer and glioblastoma report marked decreases in mammosphere/tumoursphere size and number after SFN treatment, accompanied by caspase activation and loss of stemness transcription factors, indicating that SFN not only arrests self-renewal but actively promotes CSC cell death [111,112]. SFN also modulates niche-relevant signaling that sustains CSC plasticity. Notch pathway suppression by SFN has been documented in pancreatic and other contexts, where downregulation of Notch-1 and consequent attenuation of NF-κB downstream activity reduces clonogenicity and pro-survival cytokine signaling in CSCs. This multi-node inhibition, developmental (Wnt/Notch), inflammatory (NF-κB) and epigenetic, helps explain SFN’s capacity to collapse compensatory loops that normally protect CSCs from single-agent interventions [15,113].

Clinically and translationally important is SFN’s ability to potentiate standard chemotherapies by targeting the CSC compartment. Preclinical combination studies show that SFN enhances the apoptotic and anti-clonogenic effects of taxanes and nucleoside analogues: co-treatment with SFN and docetaxel produces significantly greater apoptosis and loss of clonogenicity than either agent alone in prostate models, and reports in non-small cell lung cancer indicate that SFN augments gemcitabine activity while suppressing TGF-β/TNF-α–driven CSC enrichment. These data support SFN’s use as an adjuvant to deplete therapy-resistant CSCs and reduce relapse potential [114,115]. Collectively, an expanding literature, comprising mechanistic interrogations, spheroid and side-population assays, and in vivo validations, positions sulforaphane among the most promising anti-CSC phytochemicals. Its simultaneous targeting of Wnt, NF-κB and Notch, capacity to induce apoptosis in stem-like cells, and ability to sensitize CSCs to docetaxel and gemcitabine provide a strong translational rationale for further clinical development, particularly using well-characterized broccoli-sprout extracts or optimized SFN formulations to overcome pharmacokinetic limitations [113,116].

### 4.6. Withaferin A (WA)

WA, a steroidal lactone isolated from *Withania somnifera* (ashwagandha), has gained strong preclinical support as a multitargeted inhibitor of CSC programs. Mechanistic studies converge on two complementary modes of action: suppression of pro-stemness transcriptional programs, most notably STAT3 signaling, and induction of metabolic/oxidative stress that compromises mitochondrial function and promotes apoptosis in stem-like cells. Several recent reviews and experimental reports document that WA inhibits STAT3 phosphorylation and downstream survival gene expression while increasing ROS and activating caspase cascades, producing rapid loss of viability in CSC-enriched fractions and sensitizing cells to other cytotoxic stresses [117]. Functionally, WA reduces tumorsphere and colony formation at low-micromolar concentrations and decreases the tumor-initiating capacity of residual cells in vivo. In breast cancer models, treatment with WA (dose range tested in multiple studies typically 1–10 µM) markedly reduced colony and sphere formation and abrogated clonogenic outgrowth in serial propagation assays; similar anti-tumorsphere activity and reductions in CSC marker–positive subpopulations have been reported in lung and melanoma systems, where WA also demonstrated selective cytotoxicity toward malignant versus non-malignant cells. These functional readouts are consistent across independent laboratories and formulations, and several studies showed that WA-exposed spheres display increased apoptosis (cleaved caspase-3/PARP) and reduced expression of stemness markers such as CD44 [118,119].

WA additionally impairs metastatic phenotypes and EMT-associated programs that underlie CSC plasticity. Mechanistic work shows WA destabilizes intermediate-filament dynamics (for example vimentin), suppresses EMT transcription factors and attenuates migration/invasion in vitro, translating into decreased metastatic seeding in orthotopic and experimental metastasis models of breast cancer and melanoma. Combination approaches further accentuate these effects: recent studies report that WA combined with other epigenetic or dietary agent’s produces synergistic suppression of invasion, EMT marker expression and CSC-like behavior, highlighting WA’s capacity to both prevent dissemination and deplete the stem-like cells that seed metastases [118,120]. Finally, translationally relevant work emphasizes WA’s pleiotropic network effects, STAT3 inhibition, ROS induction, and metabolic reprogramming (including reductions in glycolytic enzyme expression and ATP production), as mechanistic pillars that together collapse the survival scaffolds of CSCs. Because these mechanisms act at multiple nodes of stemness and stress tolerance, WA shows consistent activity against breast, lung and melanoma CSC models and warrants further development with attention to optimized delivery and safety profiling for clinical translation.

### 4.7. Quercetin

**Figure 4:**
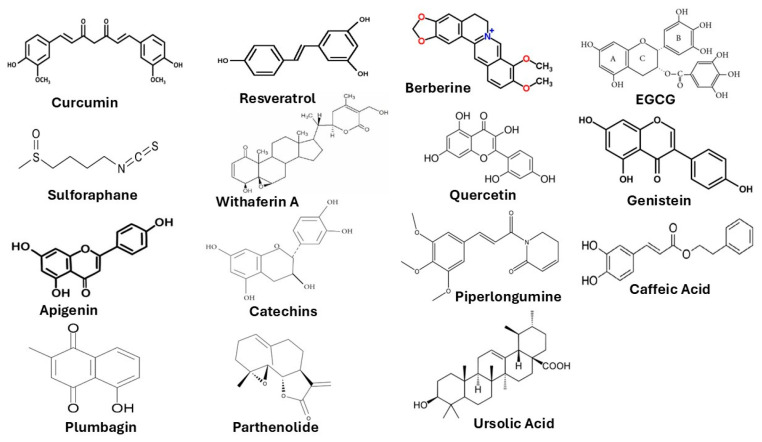
Chemical structures of representative bioactive phytochemicals with reported anticancer and chemopreventive properties. The figure illustrates the molecular structures of curcumin, resveratrol, berberine, EGCG, sulforaphane, withaferin A, quercetin, genistein, apigenin, catechins, piperlongumine, caffeic acid, plumbagin, parthenolide, and ursolic acid. These phytochemicals are known to modulate multiple cellular signaling pathways involved in oxidative stress, inflammation, cell proliferation, apoptosis, EMT, and cancer stem cell maintenance, highlighting their therapeutic potential in cancer prevention and treatment.

Quercetin, a widely distributed flavonol found in onions, apples and many medicinal plants, has emerged from recent preclinical work as a pleiotropic inhibitor of CSC programs through coordinated suppression of survival signaling, promotion of apoptosis and blockade of EMT-driven plasticity. Multiple mechanistic studies and contemporary reviews document that quercetin attenuates PI3K/Akt signaling and downstream mTOR activity in CSC-enriched populations, leading to reduced phosphorylation of Akt and diminished expression of prosurvival effectors; this molecular signature is tightly associated with loss of clonogenicity and reduced mammosphere/tumorsphere formation in BC models [121,122]. Beyond PI3K/Akt, quercetin interferes with Wnt/β-catenin signaling nodes that are fundamental to stemness maintenance. In colorectal and liver cancer contexts, quercetin treatment reduces nuclear β-catenin accumulation and downregulates Wnt target genes, effects that translate into fewer and smaller spheroids, diminished expression of stem markers and loss of tumor-initiating capacity in orthotopic assays reported in the recent literature. These results indicate that quercetin targets both upstream survival cues and core developmental programs that sustain CSC self-renewal [30,123]. Quercetin also promotes apoptotic clearance of stem-like cells via p53-dependent mechanisms and modulation of pro- and anti-apoptotic regulators. Experimental studies report quercetin-induced up-regulation of p53 and downstream effectors (for example p21 and Bax), with concurrent decreases in Bcl-2 and survivin expression, producing increased caspase activation in CSC-enriched fractions and enhanced chemosensitivity. These pro-apoptotic effects have been observed across breast and prostate models, where quercetin not only reduces viable CSC numbers in vitro but also diminishes the capacity of residual cells to reinitiate tumors in vivo [124,125]. Figure 4 shows the chemical structures of representative bioactive phytochemicals with reported anticancer and chemopreventive properties.

**Figure 5:**
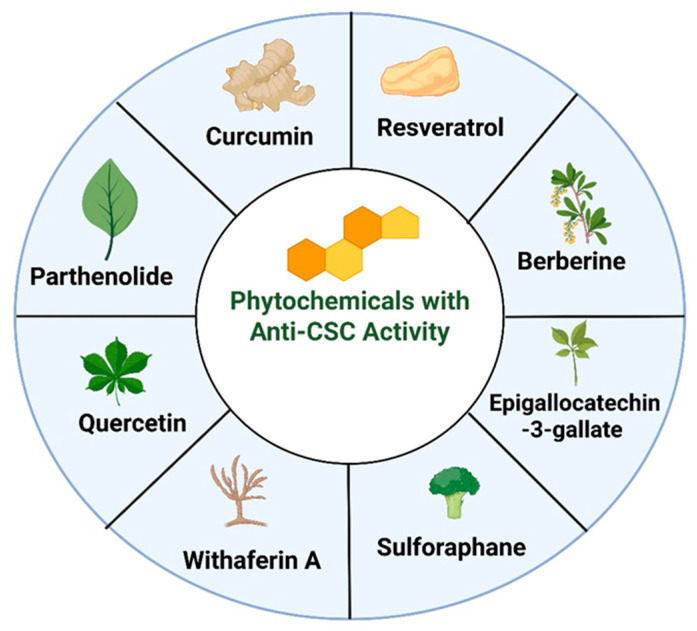
Major phytochemicals with demonstrated anti-CSC potential. These bioactive compounds target CSC-associated mechanisms including Wnt/β-catenin, Notch, Hedgehog, STAT3, NF-κB, and PI3K/Akt signaling, thereby reducing CSC maintenance, invasion, and therapy resistance.

Importantly, quercetin impairs EMT programs that promote CSC plasticity and metastatic competence. Recent work shows that quercetin downregulates EMT transcription factors and mesenchymal markers (including Snail, vimentin and Twist), restores epithelial marker expression and suppresses migration/invasion in multiple tumor systems; these EMT-reversing actions correlate with decreases in CD44^+^/CD24^−^ or CD44^+^/CD133^+^ CSC subpopulations and with markedly reduced metastatic seeding in preclinical models of liver and prostate cancer [126,127]. Collectively, the contemporary evidence base supports quercetin as a multi-modal anti-CSC phytochemical: it inhibits PI3K/Akt and Wnt signaling, enhances apoptosis through p53 activation and related pathways, and blocks EMT-driven plasticity, mechanisms that have produced consistent CSC reductions in breast, prostate and liver cancer models. Although limitations in bioavailability remain a translational challenge, recent advances in nanoformulations and combinatorial regimens have improved quercetin’s delivery and anti-CSC efficacy, making it a promising candidate for further preclinical development and rational combination with standard therapies [123,128]. Figure 5 provides an overview of the major phytochemicals with demonstrated anti-CSC potential. Table 1 summarizes the major phytochemicals and their anti-CSC mechanisms.

## 5. Synergistic Effects with Chemotherapy

Phytochemicals consistently enhance the efficacy of standard chemotherapeutics by acting on the therapy-resistant CSC compartment through several complementary mechanisms. Mechanistically, many plant-derived compounds sensitize CSCs to cytotoxic drugs (paclitaxel, cisplatin, doxorubicin, 5-fluorouracil) by inhibiting prosurvival signaling (STAT3, PI3K/Akt, NF-κB, Wnt/β-catenin), lowering the threshold for apoptosis, and collapsing the metabolic programs that sustain quiescent, drug-tolerant CSCs. These molecular actions translate into reduced tumorsphere formation, decreased tumor-initiating cell frequency after treatment, and improved chemotherapy-induced cell death in multiple preclinical models. For example, sulforaphane combined with taxanes produces greater apoptosis and deeper depletion of breast CSCs than either agent alone, supporting SFN as an effective CSC-targeting chemosensitizer [132]. A second, well-documented mechanism is suppression of multidrug resistance (MDR) proteins. Phytochemicals such as EGCG, resveratrol and curcumin downregulate or inhibit ABC transporter family members (ABCB1/P-gp, ABCG2/BCRP, ABCCs) that mediate drug efflux from CSCs, thereby increasing intracellular drug accumulation and restoring cytotoxic activity against previously refractory cells. Reviews and mechanistic studies show that targeting ABC transporters with natural compounds reverses side-population/CSC phenotypes and is a major route by which phytochemical–chemotherapy combinations overcome MDR [142,143]. These molecular effects manifest as clinically relevant outcomes in preclinical and early clinical work: combinations reduce tumor recurrence and metastatic spread in animal models and, in some cases, improve objective responses in patients. Curcumin plus paclitaxel has been tested in clinical settings with improved response and tolerability reported in metastatic breast cancer cohorts, and multiple preclinical studies and meta-analyses support the ability of curcumin to reverse paclitaxel resistance and diminish residual CSC fractions. Similarly, resveratrol enhances 5-FU efficacy against colorectal CSC-like cells and reduces migration/invasion after chemotherapy, while EGCG co-treatment augments cisplatin cytotoxicity and reduces CSC markers in lung and gastrointestinal models. These examples demonstrate that phytochemical co-treatments can achieve deeper, more durable tumor control by specifically targeting the CSC reservoir that seeds relapse [144,145,146].

An additional translational advantage is toxicity modulation. Several phytochemicals show protective effects on normal tissues or reduce chemotherapy-related adverse effects while preserving (or enhancing) anticancer activity; resveratrol and curcumin have both been reported to attenuate certain drug toxicities (for example cardiotoxicity and fatigue) in preclinical and early clinical studies, suggesting that phytochemical adjuvants may widen therapeutic windows when used rationally with standard agents. Taken together, the preclinical and emerging clinical literature supports specific, evidence-based combinations with demonstrated anti-CSC benefit, curcumin + paclitaxel, sulforaphane + docetaxel (or paclitaxel), resveratrol + 5-FU, and EGCG + cisplatin, each of which has shown synergistic activity in reducing CSC markers, restoring chemosensitivity, lowering recurrence in animal models and, in some cases, improving patient-level outcomes [114,144]. While promising, these data also underscore challenges: variability in phytochemical formulation and bioavailability, context-dependent effects across tumor types and doses, and occasional antagonism reported in heterogenous cell lines. Therefore, the most robust preclinical and translational successes combine mechanistic research (to match phytochemical action to tumor biology), optimized delivery (nanoformulations, prodrugs), and rational trial designs that measure CSC endpoints (tumorsphere assays, ALDH/marker frequency, minimal residual disease) alongside conventional clinical outcomes. When so deployed, phytochemical–chemotherapy combinations offer a pragmatic, biologically grounded route to deplete CSCs, reduce metastasis and recurrence, and potentially lower the toxicity burden of cancer treatment [142,147]. Table 2 presents the phytochemical–chemotherapy synergy matrix.

## 6. Nanotechnology-Enhanced Delivery of Anti-CSC Phytochemicals

While many phytochemicals exhibit potent anti CSC activity in vitro, their translation into effective therapies has been hindered by poor aqueous solubility, rapid systemic clearance, and limited tumor bioavailability. Nanotechnology-based delivery systems, including liposomes, polymeric nanoparticles (NPs), nano phytosomes, solid lipid nanoparticles (SLNs), nanostructured lipid carriers (NLCs), and even metal (e.g., gold) nanoparticle conjugates, have emerged as powerful platforms to overcome these physicochemical and pharmacokinetic barriers, and to preferentially deliver phytochemicals to tumor and CSC niches. Recent comprehensive reviews categorize these nanocarriers into distinct classes based on composition, structural organization, and mechanism of delivery: (i) lipid-based systems (liposomes, SLNs, NLCs, nano-phytosomes, nanoemulsions); (ii) polymeric carriers (biodegradable polymeric nanoparticles, polymer-based nanocapsules, polymeric micelles); and (iii) inorganic or hybrid systems (metal nanoparticles such as gold or mesoporous silica, and lipid–polymer hybrid nanoparticles). Each class exhibits unique benefits for enhancing stability, bioavailability, controlled release, and both passive and active tumor/CSC targeting. These classifications reflect the broad consensus in current nanoparticle-phytochemical research and reviews in the fields [161,162,163,164].

Recent comprehensive reviews highlight that nanoformulations of phytochemicals such as curcumin, quercetin, resveratrol, and others consistently achieve 3–10-fold improvements in cellular uptake, solubility, stability, and targeted tumor accumulation compared with free compounds [165,166,167]. For example, encapsulating curcumin in polymeric nanoparticles (e.g., PLGA based NPs) dramatically enhances its cytotoxicity relative to free curcumin, owing to vastly improved cell uptake and intracellular delivery [91,168]. Lipid-based carriers are among the most widely investigated platforms for phytochemical delivery. Liposomes, composed of phospholipid bilayers, encapsulate hydrophilic molecules in their aqueous core and hydrophobic agents within the lipid bilayer, offering high biocompatibility, low toxicity, and the capacity for surface modification (e.g., PEGylation or ligand conjugation) to facilitate active targeting mechanisms. Liposomal formulations of resveratrol and quercetin have demonstrated improved cellular uptake and enhanced anticancer efficacy compared with their free counterparts. SLNs and NLCs, constructed from solid or mixed solid–liquid lipids stabilized by surfactants, provide improved physicochemical stability, controlled release, high drug loading capacity, and protection against degradation, features particularly advantageous for unstable phytochemicals such as curcumin and EGCG. NLCs, with their imperfect lipid matrix, further enhance drug encapsulation efficiency and minimize leakage relative to SLNs [164,169]. Similarly, curcumin-loaded solid lipid nanoparticles (Cur SLNs) display higher stability, controlled release, and superior cytotoxicity in breast cancer cells compared to free curcumin; in one study, the IC_50_ for Cur SLNs was significantly lower than for free curcumin, indicating enhanced potency [170,171]. As an illustration of enhanced anti CSC function, hybrid lipid nanocapsules loaded with curcumin (CMN-nHLCs) reduced mammosphere formation and stemness in breast cancer stem-like cells about 2.5-fold more effectively than free curcumin [171].

Polymeric nanoparticles, fabricated from biodegradable polymers such as PLGA, PLA, chitosan, or PEGylated polymers, allow precise control over particle size, surface charge, and drug release kinetics. They also confer protection of labile phytochemicals from enzymatic degradation and improve systemic circulation times. Multiple studies have shown enhanced anti-tumor and anti-CSC activity of phytochemical-loaded polymeric nanocarriers, exemplified by quercetin and resveratrol encapsulated in polymer-based systems with superior in vitro and in vivo efficacy relative to free phytochemicals [172]. Beyond simply increasing solubility or uptake, nanocarriers also enable better tumor targeting, controlled drug release, and preferential accumulation in CSC rich microenvironments. Lipid-based carriers such as SLNs or nanostructured lipid carriers exploit the enhanced permeability and retention (EPR) effect in tumors and can be further modified (e.g., with surface ligands) to actively target tumor cells [173,174]. Polymeric nanocarriers offer additional advantages: controlled, sustained release; protection of labile phytochemicals; and minimization of off-target toxicity [91,175]. Inorganic and hybrid nanocarrier platforms expand the toolkit for anti-CSC phytochemical delivery. Gold nanoparticles conjugated to resveratrol or other phytochemicals provide high surface-to-volume ratios, facile surface functionalization, and unique optical properties for theranostic applications. Resveratrol-capped gold nanoparticles have been shown to more effectively inhibit cancer cell proliferation and modulate key signaling pathways (e.g., NF-κB, Akt) compared with free resveratrol. Mesoporous silica nanoparticles, another class of inorganic carriers, offer tunable pore structures and large surface areas to load and release phytochemicals with enhanced tumor uptake and cytotoxicity. Hybrid lipid–polymer nanoparticles combine the stability and controlled release of polymeric systems with the biocompatibility and targeting capacity of lipids, demonstrating synergistic benefits for both pharmacokinetics and bioactivity [176,177].

Moreover, nanotechnology-based delivery is not limited to curcumin. Emerging studies demonstrate that other phytochemicals, such as resveratrol and quercetin, also benefit from nano encapsulation, for instance, resveratrol-conjugated gold nanoparticles enhance cellular uptake and cytotoxicity in various cancer cells compared to free resveratrol, while nano formulations of quercetin improve solubility, stability, and anti-cancer efficacy [177]. Taken together, these advances support the notion that nanotechnology-enhanced delivery of phytochemicals is not simply a technical improvement, but a fundamental enabler of translating phytochemical-based CSC–targeting into realistic therapeutic strategies. By improving solubility, stability, bioavailability, tumor targeting and controlled release, and by increasing accumulation in CSC rich niches, nanoformulations significantly amplify the anti CSC efficacy of phytochemicals, and lower the systemic toxicity and dosing burden. As these formulation technologies mature, they may bridge the gap between in vitro promise and in vivo applicability, making phytochemical-based CSC therapies a viable component of future cancer treatment paradigms. Table 3 outlines nanoformulations developed to enhance phytochemical pharmacokinetics and anti-CSC activity.

## 7. Preclinical Evidence Supporting Anti-CSC Effects of Phytochemicals

An expanding body of preclinical research robustly supports that selected phytochemicals, notably curcumin, EGCG, sulforaphane, resveratrol, withaferin A, berberine, and quercetin, possess multi-faceted anti-CSC activity across diverse cancer models. These compounds frequently exert their anti-CSC effects by modulating key self-renewal and survival pathways such as Wnt/β-catenin, Notch, Hedgehog, PI3K/Akt, STAT3, and NF-κB, thereby reducing CSC proliferation, stemness marker expression, and tumorigenic potential while enhancing apoptosis and inhibiting metastasis [15,16]. In preclinical cell culture and xenograft models, curcumin has been repeatedly shown to suppress CSC phenotypes by down-regulating key stemness pathways such as Wnt/β-catenin, PI3K/AKT, Notch, and STAT3, thereby reducing expression of CSC surface markers (e.g., ALDH, CD44, CD133) and decreasing self-renewal and survival of CSCs across various tumor types. Curcumin also modulates microRNAs that regulate CSC maintenance and sensitizes CSCs to chemotherapeutic agents in preclinical models of breast and other cancers [189]. EGCG from green tea exhibits anti-CSC effects in prostate and other models by inhibiting spheroid/mammosphere formation, inducing apoptosis in CSCs, and blocking EMT and survival signaling; in some systems, EGCG synergizes with quercetin to more effectively reduce CSC self-renewal and invasive properties [190]. Sulforaphane, a cruciferous vegetable isothiocyanate, has demonstrated capacity to markedly reduce CSC populations, especially in breast cancer models, by reversing chemotherapy-induced enrichment of ALDH-positive cells and significantly decreasing primary and secondary mammosphere formation in vitro and tumor formation in vivo [162].

Resveratrol has been shown in multiple preclinical systems to suppress mammosphere formation, reduce CSC proliferation, and disrupt survival and stemness pathways such as Wnt/β-catenin and NF-κB. It also enhances apoptotic cascades through caspase activation, decreases expression of anti-apoptotic proteins (e.g., Bcl-2, XIAP), and, in combination with chemotherapy, reverses drug resistance in CSC-enriched preclinical models [127]. Withaferin A, a steroidal lactone from *Withania somnifera*, has been validated in preclinical lung and GI cancer CSC models to inhibit sphere formation, reduce the percentage of side-population CSCs, and induce apoptosis via STAT3/mTOR axis suppression; in other systems, withaferin A down-regulates Notch signaling and stemness markers (e.g., CD44, Oct4) and blocks metastatic CSC formation in xenograft models [191]. Berberine, an isoquinoline alkaloid, shows strong anti-CSC effects in both in vitro CSC cultures and in vivo xenograft models such as oral squamous carcinoma. Its dose-dependently reduces ALDH1 activity, self-renewal, colony formation, migration, and invasion of CSCs, suppresses oncogenic miR-21, and attenuates tumor growth in mouse xenograft, also potentiating chemotherapeutic efficacy [192]. Quercetin has demonstrated inhibitory effects on CSC characteristics in preclinical studies, including decreased proliferation, invasion, self-renewal, and β-catenin signaling in pancreatic CSC models, with enhanced sensitivity to chemotherapy when combined with conventional drugs [193]. Collectively, these preclinical datasets across in vitro assays, mammosphere/tumorsphere formation, CSC marker profiling, pathway modulation analyses, and multiple in vivo xenograft studies underline that these phytochemicals exert anti-CSC effects by disrupting self-renewal, survival signaling, and drug resistance pathways central to CSC biology, and in many cases also enhance the effectiveness of standard anticancer therapies [16]. Table 4 compares in vitro and in vivo efficacy across various CSC assay platforms.

## 8. Clinical Evaluation of Phytochemicals: Safety, Efficacy, and Translational Potential

Despite robust preclinical evidence demonstrating that phytochemicals such as curcumin, EGCG, sulforaphane, resveratrol, and genistein modulate CSC signaling pathways (e.g., Wnt/β-catenin, Notch, Hedgehog, STAT3) and attenuate stemness-associated phenotypes in vitro and in vivo, the translation of these agents into definitive CSC-targeted clinical strategies has been modest and mostly exploratory. Curcumin has the most extensive human clinical trial history among the phytochemicals of interest. Multiple phase II and phase III clinical trials have explored curcumin’s safety and potential therapeutic impact in diverse cancer contexts, including prostate, colorectal, breast, and cervical cancers. For example, a phase 3 trial (NCT03769766) is evaluating whether curcumin can prevent progression of low-risk prostate cancer under active surveillance, comparing curcumin to placebo to determine effects on disease progression and prostate-specific antigen levels. Another phase 3 trial (NCT00295035) assessed the combination of curcumin with gemcitabine and celecoxib in metastatic colon cancer patients to gather additional safety and efficacy data. Additional studies (e.g., NCT01490996) have combined curcumin with FOLFOX chemotherapy in patients with inoperable colorectal cancer to evaluate tissue biomarkers and tolerability. Other interventional trials, such as NCT03072992 in advanced breast cancer, investigated curcumin with paclitaxel to assess objective response rate and progression-free survival. Although many of these trials have primarily reported safety and tolerability, with no definitive survival or progression data widely published, curcumin has been generally well tolerated at administered doses with acceptable safety profiles and measurable biomarker modulation in cancer patients [202,203,204].

EGCG has been evaluated in human cancer preventive and therapeutic studies, albeit to a lesser extent than curcumin. A notable trial (NCT02891538) investigated the chemopreventive effects of EGCG supplementation in colorectal cancer (CRC) patients, assessing safety and potential effects on CRC progression markers. Another government-sponsored phase II trial, the CATCH-B study, is testing whether EGCG can prevent liver cancer development in individuals with cirrhosis, focusing on its ability to reduce cancer risk rather than treat established disease. These trials demonstrate that EGCG is generally safe and tolerated in human subjects at used doses, with biomarker and prevention-oriented endpoints, but clear evidence of therapeutic efficacy in reducing tumor burden or improving survival in active cancer remains limited [205,206].

Sulforaphane-rich preparations derived from broccoli or broccoli sprouts have been studied in clinical settings (e.g., breast cancer trials such as NCT00843167 and NCT00982319), focusing mainly on modulation of proliferation markers like Ki-67 rather than direct CSC endpoints, and while effects on surrogate biomarkers are reported, meaningful clinical efficacy data remain limited and inconclusive [207,208]. Notably, sulforaphane has been recognized as generally safe (often classified as GRAS) and is included in various clinical studies for other diseases, but rigorous cancer-specific clinical trials with efficacy endpoints such as progression or survival are sparse. This indicates that sulforaphane’s translation from mechanistic preclinical evidence to human cancer trials remains at an early exploratory stage [209].

Resveratrol and genistein have been the subjects of numerous clinical investigations (with ≥30 genistein trials across various cancer types and smaller resveratrol studies), but most outcomes pertain to tolerability, systemic pharmacodynamics, and adjunctive effects rather than explicit CSC eradication, and none have demonstrated definitive clinical benefits attributable specifically to CSC targeting [210]. In addition, resveratrol has been included in early phase clinical trials involving cancer patients, particularly in colorectal and prostate cancers. Registered studies like NCT00098969 and NCT00256334 assessed resveratrol in colon cancer contexts, primarily focusing on safety, tissue levels, and biomarker changes rather than definitive clinical outcomes. These trials confirmed that resveratrol can be administered safely at high oral doses, but systemic bioavailability limitations and rapid metabolism are consistent challenges that have impeded demonstration of significant anticancer efficacy in human studies. Thus, while resveratrol’s safety profile in cancer patients appears acceptable, the translational impact on meaningful patient-centric outcomes remains inconclusive [211].

For withaferin A, direct clinical trial evidence in cancer patients is just emerging. The recently registered phase I/II clinical trial NCT05610735 is evaluating the tolerability and preliminary efficacy of an Ashwagandha extract containing withaferin A in combination with liposomal doxorubicin for recurrent ovarian cancer. This study’s goals include determining dose escalation safety and responses such as partial and complete responses or stable disease, but results from this trial are still pending, and no cancer outcome data have yet been published. As such, withaferin A’s clinical translation in oncology remains at an early stage, with current human studies focused on safety and feasibility [211,212].

Moreover, Berberine’s clinical evaluation in cancer patients is at an earlier stage compared with curcumin or EGCG. Some clinical investigations are examining berberine as an adjunct to chemotherapy in gastrointestinal cancers such as colon and lung cancer, with trials designed to assess safety and potential synergistic effects with conventional chemotherapeutics. Existing human studies outside oncology (e.g., metabolic disease) support berberine’s acceptable safety profile, but large, well-controlled cancer trials with efficacious outcomes are still needed to confirm translational benefit [213]. Furthermore, Quercetin has been evaluated in a limited number of clinical trials for its tolerability and biological effects, but high-quality evidence for anticancer efficacy in humans is minimal. Trials involving quercetin often focus on its antioxidant properties or general health effects, with few studies directly demonstrating tumor modulation or improved clinical outcomes in cancer patients. Like other phytochemicals, quercetin’s clinical potential is constrained by low bioavailability and requires further investigation with optimized delivery strategies [214,215].

Importantly, while none of these phytochemicals has yet been approved for cancer therapy, the overall pattern in high-impact clinical research suggests feasibility in terms of safety and biological activity, but also highlights significant translational barriers, particularly poor bioavailability, variable pharmacokinetics, and a lack of validated CSC-specific clinical endpoints, that must be addressed. Combinatorial strategies using phytochemicals with established cancer drugs and advanced delivery systems (e.g., nanoformulations) are under active investigation to overcome these limitations and enhance clinical relevance [12,216,217]. Overall, current clinical trial evidence supports the human feasibility of phytochemical-based approaches in oncology, yet rigorous, CSC-focused clinical end points and optimized formulations are essential future directions to unlock their therapeutic potential. Table 5 presents key clinical and translational data relevant to phytochemical-based CSC targeting.

## 9. Safety, Toxicity, and Dosage Considerations of Phytochemicals in Clinical Applications

In cancer research, phytochemicals have been investigated not only for anticancer efficacy but also for their safety, tolerability, and dose limits in human subjects. The most extensive clinical evidence exists for curcumin, which has been evaluated in multiple randomized and placebo-controlled trials in prostate cancer and other malignancies. For example, an RCT in men with intermittently treated prostate cancer demonstrated that daily oral curcumin suppressed PSA elevation and was well tolerated without significant adverse reactions compared with placebo over six months of administration, indicating a favorable safety profile at clinically relevant doses [228]. A separate double-blind study using 3 g/day of curcumin for up to 12 months in patients with familial adenomatous polyposis showed safety and modulation of biomarkers of carcinogenesis, reinforcing tolerability over prolonged administration [229]. In clinical trials of green tea catechins containing EGCG, such as those using Polyphenon E in men at high risk of prostate cancer, doses up to 800 mg EGCG daily were well tolerated with no significant liver enzyme elevations, while showing biochemical modulation of PSA and oxidative DNA damage markers, positioning EGCG as a relatively safe phytochemical in oncology contexts [230]. Sulforaphane, delivered as stabilized broccoli sprout extract in randomized, placebo-controlled trials in patients with rising PSA following radical prostatectomy, prolonged PSA doubling time without adverse side effects, illustrating clinical tolerability of 60–200 µmol/day regimens over several months [231].

For resveratrol, although direct phase III cancer outcome data are limited, micronized resveratrol formulations in colorectal cancer patients have increased apoptotic marker expression in malignant hepatic tissue with no serious toxicity reported, and small trials in prostate cancer have documented metabolic modulation without adverse safety concerns, supporting its acceptable safety profile at doses up to 1 g/day in oncological studies [232]. In contrast, withaferin A, a withanolide from Withania somnifera, has minimal clinical trial data in oncology; a Phase I dose-escalation study in osteosarcoma patients reported no grade 3–4 toxicities, with grade 1–2 events such as transient liver enzyme elevations, fatigue, rash, and diarrhea, but detectable systemic levels were low and comprehensive safety profiles remain preliminary [233]. Sub-acute toxicity studies in animal models support a high no-observed adverse effect level (NOAEL), yet translation of these findings to humans requires further controlled trials [234]. Berberine has demonstrated strong anticancer mechanisms in preclinical models, but human clinical trials in cancer patients are sparse; existing evidence for safety mainly derives from non-oncology settings where doses up to 1 g/day were well tolerated, yet oncology-specific safety and optimal dosing require targeted investigation before clinical recommendations [231]. Similarly, quercetin’s clinical evaluation in cancer has been limited, with most human evidence coming from metabolic and antioxidant studies; its low systemic bioavailability and lack of dedicated cancer trials necessitate further phase I/II testing to characterize safe dosage ranges and long-term effects [235]. Collectively, current cancer related studies indicate that curcumin, EGCG, sulforaphane, and resveratrol can be administered safely at defined doses with manageable side effect profiles in selected cancer populations, typically exhibiting mild gastrointestinal or biochemical changes without severe toxicity. However, withaferin A, berberine, and quercetin lack robust oncology clinical safety data, underscoring the need for well-designed phase I/II cancer trials to establish their maximum tolerated doses, organ-specific toxicities, potential herb–drug interactions, and long-term risk profiles before integration into standardized cancer care pathways [236,237].

## 10. Challenges and Limitations

Despite the encouraging preclinical evidence supporting phytochemicals as anti CSC agents, several fundamental obstacles currently hinder their translation into effective, widely adopted cancer therapies. One of the most persistent and well documented challenges is poor bioavailability. Many phytochemicals, notably hydrophobic compounds such as Curcumin and Resveratrol, suffer from low aqueous solubility, rapid first pass metabolism, and unstable systemic exposure after oral administration, limiting their capacity to reach effective concentrations in tumors or CSC niches [238,239,240]. As a result, the high concentrations used in cell-based assays often are not achievable in vivo, raising concerns about the physiologic relevance of many reported anti CSC effects [239,240]. A second major limitation stems from the lack of standardized extraction, purification, and formulation protocols for phytochemicals. The chemical composition of plant-derived products can vary significantly depending on plant variety, cultivation conditions, harvesting time, and extraction procedures [241]. This intrinsic variability complicates reproducibility across studies and undermines the ability to compare findings or aggregate data in meta-analyses. For meaningful clinical translation, rigorous standardization of raw materials, quality control, and dosing regimens is required.

Relatedly, limited clinical trial data remains a critical barrier. Although many phytochemicals have demonstrated potent CSC targeting or chemo sensitizing effects in vitro and in animal models, few have been tested in rigorous, large-scale clinical trials. Early trials (for example with curcumin or green tea polyphenols) often report inconsistent or modest therapeutic benefit, possibly due to pharmacokinetic shortcomings, variable formulations, or insufficient systemic exposure [12,242]. As a result, despite decades of preclinical research, strong evidence from randomized controlled trials supporting phytochemical based CSC therapies is still lacking. Moreover, variability among plant chemotypes and preparations adds another layer of complexity. Two extracts nominally from the same botanical species may differ dramatically in their phytochemical profile depending on cultivar, soil, climate, or processing, leading to inconsistent bioactivity even under similar experimental protocols [243]. This heterogeneity undermines both reproducibility and safety, and makes it difficult to establish standardized, clinically relevant dosing.

Finally, there is a non-negligible risk of herb–drug interactions when phytochemicals are co administered with conventional chemotherapeutic agents or other medications. Many phytochemicals influence drug metabolizing enzymes (e.g., CYPs, UGTs) or membrane transporters, potentially altering the pharmacokinetics or toxicity profile of co administered drugs [244,245]. In oncology contexts, where patients often receive complex, multitarget regimens, such interactions could unpredictably affect efficacy or safety, especially in long-term adjuvant settings. Given these challenges, it is clear that large-scale, methodologically rigorous clinical trials are needed to confirm therapeutic efficacy, define optimal formulations and dosing, and assess long-term safety of phytochemical-based regimens. Until such data are available, enthusiasm should be tempered, and translational efforts should proceed with careful design, rigorous quality control, and realistic expectations.

## 11. Future Directions

Translational progress in phytochemical-based CSC therapy will require deliberate, multidisciplinary strategies that move beyond single-agent, in vitro observations toward integrated, target-driven interventions. One high-priority avenue is multi-targeted combination therapy that pairs phytochemicals with immunotherapy and other modalities. Phytochemicals can modulate tumor-intrinsic immune evasion mechanisms and the inflammatory milieu that supports CSCs, and several recent reviews argue that combining selected natural compounds with immune checkpoint inhibitors or adoptive-cell approaches may both sensitize CSCs and reshape the tumor microenvironment to permit durable immune control. Early preclinical data support synergy between phytochemical modulation of cytokine/STAT3–NF-κB circuits and enhanced anti-tumor immunity, suggesting this is a tractable path for next-generation combination trials [143,246]. Computational triage and AI-driven discovery of plant metabolites should be brought to scale to prioritize candidates with the highest likelihood of anti-CSC activity and favorable pharmacokinetics. Advances in graph neural networks, molecular embeddings and network-pharmacology approaches now allow rapid in silico screening of large natural-product libraries, prediction of likely targets and mechanism-of-action hypotheses, and rational design of synergistic mixtures, substantially accelerating hit-to-lead workflows compared with classical bioassay-first approaches. Coupling AI predictions with metabolomics and high-throughput phenotypic screens will allow prioritization of understudied chemotypes for focused validation [247,248].

Robust genetic validation of targets in physiologically relevant models is essential: CRISPR-based functional genomics can identify CSC dependencies, validate phytochemical targets, and reveal mechanisms of resistance or compensatory signaling. Genome-wide and focused CRISPR screens have already uncovered metabolic and survival vulnerabilities specific to CSCs, and CRISPR-engineered isogenic cell lines or organoids provide powerful platforms for testing whether candidate phytochemicals act on the intended node in a human-relevant context. Integrating CRISPR perturbation data with single-cell readouts will be particularly valuable for resolving heterogeneous CSC states and for predicting which combinations (for example phytochemical + targeted inhibitor) will most effectively collapse CSC pools [249,250]. Exploration of understudied traditional medicinal plants and their chemotypes remains a fertile discovery space, especially when informed by ethnopharmacology, metabolomics and AI prioritization. Standardized phytochemical profiling (metabolomics) and rigorous bioactivity-guided fractionation can reveal novel scaffolds or multi-component synergies that single-compound screens miss; several recent efforts emphasize that integrating traditional knowledge with modern analytical tools yields higher-quality leads with clearer mechanistic hypotheses. Such discovery work must be paired with strict standardization and quality control to avoid the reproducibility problems that have hampered past translational efforts [127,248].

Finally, the deliberate development of nano-encapsulated phytochemical cocktails offers a practical route to overcome pharmacokinetic limitations and to deliver multi-agent combinations directly to CSC niches. Nanoformulations, including liposomes, polymeric nanoparticles, nano-phytosomes, solid-lipid nanoparticles and functionalized gold nanoparticles, improve solubility, stability, controlled release and tumor/CSC accumulation, enabling lower doses and reduced systemic toxicity. Preclinical reports show markedly improved cellular uptake and anti-CSC potency for nano-encapsulated phytochemicals versus free compounds, and co-encapsulation enables synchronized delivery of mechanistically complementary agents (for example apoptosis inducer + ABC-transporter inhibitor) to the same cell. The next step is to translate these platforms into rigorously designed early-phase trials that measure CSC-centric endpoints (tumorsphere frequency, ALDH/marker dynamics, MRD) alongside conventional safety and efficacy measures [161,251]. In sum, the most promising future path combines AI-guided discovery, CRISPR-validated targets, ethnobotanical breadth, immunotherapy pairing and nanotechnology-enabled delivery. By aligning these tools with standardized materials, robust preclinical models, and CSC-focused clinical endpoints, the field can move from promising preclinical signals toward reproducible, patient-level benefits. Large, carefully controlled translational studies, informed by the approaches above, are now the critical next step [147,149,161,252].

## 12. Conclusions

Phytochemicals have emerged as compelling modulators of cancer stem cell biology, offering multi-targeted mechanisms that disrupt self-renewal programs, reverse EMT phenotypes, and sensitize resistant tumor subpopulations to conventional therapy. Evidence across diverse malignancies demonstrates that compounds such as curcumin, resveratrol, berberine, EGCG, sulforaphane, withaferin A, and quercetin interfere with key signaling axes, including Wnt/β-catenin, PI3K/Akt, STAT3, NF-κB, Hedgehog, and Notch, while simultaneously downregulating established CSC markers and impairing sphere formation. Their ability to potentiate chemotherapeutic efficacy and reduce multidrug resistance underscores their potential as indispensable adjuncts to existing treatments. Despite the strength of preclinical data, translation to clinical utility remains constrained by poor bioavailability, chemotype variability, and limited trial readiness. Advances in nanotechnology have addressed many pharmacokinetic challenges, with nanoformulations markedly improving solubility, tumor targeting, and CSC-specific accumulation. Emerging opportunities, such as AI-guided phytochemical discovery, CRISPR-based mechanistic validation, and systematic exploration of traditional medicinal plants, are poised to accelerate the development of next-generation CSC-targeted therapeutics. Overall, the integration of well-characterized phytochemicals with modern drug delivery systems and rational combination strategies presents a promising path toward more durable cancer control. Realizing this potential will require rigorous standardization, mechanistic clarity, and clinically focused translational studies designed around CSC endpoints. Through these coordinated efforts, phytochemical-based interventions may evolve into clinically actionable strategies capable of mitigating relapse, metastasis, and therapy resistance.

## Figures and Tables

**Table 1:** Summary of major phytochemicals and anti-CSC mechanisms.

Phytochemical (Common + Chemical Name)	Source (Plant)	Reported Anti-CSC Mechanisms	Key CSC Assays Used	Cancer Types Reported	References
Curcumin (diferuloylmethane)	*Curcuma longa* (turmeric)	Inhibits Wnt/β-catenin and Sonic-hedgehog signaling, downregulates CSC markers (CD44, ALDH), reverses EMT and reduces self-renewal.	Tumorsphere formation, ALDEFLUOR/ALDH activity, CD44/CD24 flow cytometry, xenograft tumorigenicity assays.	Breast, lung, colorectal, pancreatic.	[111,129]
Resveratrol (3,5,4′-trihydroxy-trans-stilbene)	*Vitis vinifera* (grape), peanuts	Suppresses STAT3/JAK and Src pathways, decreases ALDH and CD44^+^ populations, inhibits EMT and colony/sphere formation, promotes differentiation/apoptosis of CSCs.	Tumorspheres, ALDH, CD marker profiling, in vivo xenografts.	Lung, breast, colorectal, ovarian.	[130,131]
Sulforaphane (isothiocyanate)	Cruciferous vegetables (broccoli, *Brassica* spp.)	Blocks Wnt/β-catenin and self-renewal programs, downregulates ALDH and pluripotency factors (Nanog, Sox2), sensitizes CSCs to chemo.	Sphere formation, ALDEFLUOR, side-population, xenograft limiting-dilution assays.	Breast (including TNBC), pancreatic, prostate.	[113,132]
EGCG (epigallocatechin-3-gallate)	*Camellia sinensis* (green tea)	Inhibits Src/JAK/STAT3 and EMT programs, reduces stemness markers and sphere formation, promotes apoptosis and chemosensitization.	Tumorsphere assays, CD marker changes, colony formation, in vivo tumor models.	Ovarian, prostate, breast, colorectal.	[133,134]
Genistein (an isoflavone: 4′,5,7-trihydroxyisoflavone)	*Glycine max* (soybean)	Downregulates Hedgehog/Notch/Wnt signaling in CSC contexts, reduces ALDH activity and CD44^+^ populations, induces differentiation and apoptosis.	ALDEFLUOR, sphere formation, flow cytometry for CD markers, xenograft assays.	Breast, pancreatic, prostate.	[135,136]
Quercetin (3,3′,4′,5,7-pentahydroxyflavone)	Widely in fruits/vegetables (onion, apple, capers)	Inhibits PI3K/Akt/mTOR and NF-κB signaling, decreases CD44^+^/CD24^−^ CSC fraction, induces cell-cycle arrest and apoptosis, enhances chemosensitivity.	Sphere formation, CD44/CD24 profiling, viability/apoptosis assays, xenografts.	Breast, colon, gastric.	[15,137]
Parthenolide (sesquiterpene lactone)	*Tanacetum parthenium* (feverfew)	Inhibits NF-κB signaling, selectively targets side-population/ALDH^+^ CSCs, induces ROS-mediated apoptosis and impairs self-renewal.	Side-population assays, ALDEFLUOR, sphere assays, in vivo xenograft reduction in CSC-driven growth.	Leukemia, nasopharyngeal, solid tumors in preclinical studies.	[138,139]
Berberine (isoquinoline alkaloid)	*Berberis* spp., *Coptis chinensis*	Suppresses Hedgehog/GLI and other stemness pathways, reduces chemotherapy-enriched CSC traits (migration/invasion), downregulates CSC markers and tumorigenicity.	Sphere and migration/invasion assays, ALDH, xenograft metastasis/tumorigenicity studies.	Ovarian, breast, colon models.	[140,141]

**Table 2:** Phytochemical + Chemotherapy Synergy matrix.

Combination (Phytochemical + Chemo Drug)	Model (Cell/Animal)	Measured Synergy (CI or % Effect; Concentration/Dose)	Effect on CSC Markers	Clinical Status/Comment	References
Curcumin + Paclitaxel	Multiple cancer cell lines (breast, ovarian, lung); xenograft models reported in some studies.	Synergistic growth inhibition (CI reported <1 in multiple studies and nano-coformulation papers; example CI values 0.43–0.59 in co-loaded nanoparticle studies). Reported enhanced apoptosis and greater tumor growth suppression vs. either agent alone (in vivo dosing varied by study).	Reduced tumorsphere formation, decreased CD44^+^/ALDH^+^ fractions in CD44-high models; improved chemosensitivity of CSC-like populations.	Preclinical (extensive); at least one randomized clinical trial assessed IV curcumin + paclitaxel in advanced breast cancer showing improved ORR and tolerability signals (pilot/phase II style evidence).	[144,148,149]
Sulforaphane (SFN) + Gemcitabine	In vitro: cholangiocarcinoma and pancreatic cancer cell lines; in vivo xenografts in pancreatic/TNBC models in related literature.	Quantitative CI data reported: example from HuH28 cells—CI values 0.323–0.738 across tested SFN (8.5–34.2 µM) + GEM (0.17–0.71 µM) combinations indicating synergism at multiple dose pairs. SFN enhanced gemcitabine cytotoxicity and reduced tumor-initiating frequency in limiting-dilution assays (in vivo).	Decreased ALDH^+^ fraction, reduced sphere formation and tumor-initiating frequency; SFN sensitizes CSCs to gemcitabine and taxanes (reports in TNBC).	Preclinical strong evidence; actively pursued as a chemosensitizer in multiple preclinical programs.	[150,151]
Resveratrol + Cisplatin	In vitro (HepG2, ovarian, lung cell lines); some animal xenograft work.	Reported enhanced cytotoxicity and apoptosis vs. cisplatin alone; many studies report percent viability reductions or enhanced apoptosis markers at 10–100 µM resveratrol with cisplatin. Some studies report mechanistic enhancement (ROS, mitochondrial apoptosis). CI sometimes calculated or demonstrably synergistic depending on schedule.	Decreases in sphere formation and stemness gene expression reported in several models; combination reduces CSC-enriched subpopulations in vitro.	Preclinical; multiple in vitro and animal reports support synergy—no large randomized clinical trials combining resveratrol + cisplatin to date.	[152,153]
EGCG (green tea) + Doxorubicin (or other anthracyclines)	In vitro (breast, lung, bladder, osteosarcoma models) and in vivo xenografts.	Demonstrated synergism in multiple studies (CI < 1 or schedule-dependent potentiation). Example: EGCG + DOX yielded greater tumor growth inhibition and % viability reduction than either alone; schedule matters (pre- vs. co-treatment). Concentrations commonly tested: 5–50 µM EGCG in vitro.	Reduced tumorsphere formation and ALDH^+^/CD44^+^ fractions in some studies; inhibits stemness pathways (STAT3, NF-κB) and enhances chemo-induced apoptosis in CSCs.	Preclinical; extensive preclinical evidence and translational interest; EGCG has been tested clinically mostly as dietary supplement/adjuvant but not as formal cisplatin/DOX co-regimen in large trials.	[154,155]
Parthenolide (PTL) + Standard chemo (e.g., Doxorubicin/Ara-C)	Leukemia models (cell lines, patient-derived xenografts/AML LSC models); some solid tumor cell studies.	PTL markedly sensitizes chemo-resistant leukemia cells and LSCs; studies report greatly reduced LSC frequency and impaired secondary engraftment vs. chemo alone. CI values not always reported numerically but functional eradication of LSCs reported at low-micromolar PTL when combined with chemo.	Depletion of ALDH^+^/side-population/leukemic stem cell markers; reduced colony formation and long-term engraftment.	Preclinical strong signal for AML LSC targeting; PTL derivatives advanced into translational development (preclinical/early clinical exploration).	[156]
Quercetin + 5-Fluorouracil (5-FU)	Colon, breast, melanoma cell lines; some ovo/in vivo corroboration in preclinical models.	Enhanced cytotoxicity vs. 5-FU alone (examples: up to ~40% additional viability reduction in some models); CI values variably reported (many studies report significant sensitization).	Reversal of 5-FU resistance in colon models; decreased sphere formation/clonogenicity and downregulation of stemness-associated pathways (Nrf2/HO-1, etc.).	Preclinical (in vitro/in ovo/in vivo); mechanistic and translational interest with some rationale for trialing as adjuvant.	[157,158]
Genistein (soy isoflavone) + Gemcitabine	In vitro and in vivo xenograft pancreatic cancer models; AXP107-11 clinical development (phase I pilot/combination studies).	Preclinical: genistein pretreatment enhanced gemcitabine cytotoxicity (e.g., growth inhibition 60–80% vs. ~25–30% for gemcitabine alone in some lines). AXP107-11 (a botanical genistein formulation) reached clinical testing combined with gemcitabine showing favorable PK and tolerability in a phase I trial.	Reported reduction in CSC markers, decreased sphere formation and tumor volume in xenografts; improved chemosensitivity in resistant models.	Preclinical → early clinical (AXP107-11 + gemcitabine completed phase I/pilot studies with supportive PK/safety data).	[159,160]

**Table 3:** Nanoformulations used to improve phytochemical PK and anti-CSC outcomes.

Phytochemical + Formulation	Particle Size/Zeta Potential (Reported)	Bioavailability Improvement (Fold ↑ vs. Free; Cmax/AUC Data)	CSC Functional Outcome (Tumorsphere, Tumor Regression, LSC Ablation, etc.)	Targeting Moiety	References
Curcumin—PLGA nanoparticles (CUR-PLGA NPs)	~200 nm (spherical); (reported sizes ≈ 150–200 nm in related studies).	~5.6-fold relative oral bioavailability vs. free curcumin (rat PK study; longer t½ reported).	Improved cellular uptake and greater antiproliferative potency vs. free curcumin; enhanced in vivo tumor suppression in xenograft models and improved CSC targeting in HA- or ligand-functionalized variants.	none (basic PLGA NP); HA-conjugated variants exist for CD44 targeting.	[178,179]
Curcumin—HA-conjugated PLGA (Cur-HA-PLGA NPs)	Cur-PLGA-NPs: ~225.0 ± 3.5 nm, zeta potential −13.6 ± 0.3 mV; Cur-HA-PLGA-NPs: ~234.4 ± 1.3 nm, zeta potential −14.6 ± 0.3 mV (as directly measured in Hlaing et al., 2022).	Improved tumor accumulation and cellular uptake vs. free Cur/untargeted NP; PK improvement reported qualitatively (higher tumor levels/retention).	Potentiated depletion of CD44^+^ cell populations and enhanced tumorsphere inhibition in breast/colorectal models versus free curcumin; improved in vivo anti-tumor efficacy in CD44-high tumors.	Hyaluronic acid (HA)—targets CD44 on CSCs.	[180,181]
Parthenolide (PTL)—MSV/micelle + porous silicon MSV-PTL	Multistage vector (MSV): porous silicon microparticles (first-stage microparticle ~1–3 µm discoidal porous silicon) that carry 2nd-stage nanoparticles (nm scale).	MSV delivery increased local bone-marrow delivery of PTL (higher BM drug levels vs. micelle or free drug); plasma levels often low while tissue delivery increased—reported as enhanced target tissue exposure rather than simple AUC fold given.	Ablation of leukemia stem cells (LSCs) in AML patient-derived xenografts (reduced LSC frequency, impaired secondary engraftment); superior in vivo eradication of CSC/LSC compared with free PTL or micellar PTL.	E-selectin thioaptamer (ESTA) functionalization to direct MSV to bone-marrow endothelium.	[182,183]
Resveratrol—polymeric/lipid-polymer hybrid NPs (e.g., RSV-PCL, RSV-LPHNP)	Example reported: RSV-PCL NPs ~138.6 nm (one study); size varies with formulation (100–250 nm common).	Nanoencapsulation markedly improves circulation time and tumor delivery; specific fold-AUC depends on formulation—many reports show increased tissue exposure and improved in vivo efficacy versus free RSV.	Enhanced suppression of tumor growth in xenograft models, improved cellular uptake, and greater reduction in tumorsphere formation/CSC markers when co-delivered with chemo agents.	Some studies use TPGS or PEGylation to improve circulation/cell uptake; active ligands reported in targeted variants.	[184,185]
EGCG—lipid/polymeric nanoparticles or liposomes	Reported NP sizes typically 50–250 nm depending on carrier; many formulations report PDI < 0.3 and modest zeta potentials.	Nanoformulations increase EGCG stability (reduced degradation), extend circulation and improve cellular delivery; quantitative fold-AUC varies by system (often reported as “significantly increased” in individual studies).	Improved tumorsphere inhibition, reduced ALDH^+^/CD44^+^ fractions and enhanced chemosensitization in breast and other CSC models versus free EGCG.	PEGylation/ligand modifications used in several reports to improve tumor targeting.	[186,187]
Berberine—liquid crystalline nanoparticles (LCNs)/liposomes	Example: berberine-LCNs average particle size ~181.3 nm, high encapsulation efficiency reported; spherical morphology.	Improved cellular delivery and sustained release; PK/bioavailability improvements described qualitatively (enhanced cellular uptake and in vivo efficacy in some models).	Increased antiproliferative activity, reduced colony/tumorsphere formation and migration/invasion in vitro; some formulations show improved tumor suppression in vivo.	No targeting moiety in many reports; targeting ligands are feasible in follow-ups.	[188]

**Table 4:** Comparative in vitro/in vivo efficacy across CSC assays.

Phytochemical	Assay Type (Example Readout)	Reported Quantitative Result (Concentration/Dose)	Model (Cell Line or Animal Model)	References
Curcumin (diferuloylmethane)	Tumorsphere formation; colony formation; JAK2/STAT3 readouts	Significant inhibition of tumor-sphere formation at 20–40 µM; colony-forming activity reduced at ≈10 µM (dose-dependent).	NCI-H460 lung cancer cells (in vitro); xenograft experiments reported in the study.	[194]
Sulforaphane (SFN)	Sphere formation; ALDEFLUOR (ALDH) activity; limiting-dilution xenograft assays	Reported suppression of sphere formation and ALDH^+^ fraction at low-micromolar exposures (typical experimental ranges 1–10 µM in vitro); SFN reduced tumor-initiating frequency in limiting-dilution xenografts in several studies.	Multiple cancer cell lines (breast, pancreatic, OSCC models) and xenograft mouse models in primary studies and reviews.	[113,195]
Resveratrol (3,5,4′-trihydroxy-trans-stilbene)	Primary/secondary spheroid formation; ALDH and pluripotency factor expression; in vivo tumor growth	In vitro inhibition of primary/secondary spheroid formation and CSC marker expression at experimental concentrations commonly tested in the 10–100 µM range; resveratrol reduced CSC-driven tumor growth in Kras^G12D mouse and human CSC xenograft models (oral or dietary dosing regimens used in vivo).	Human pancreatic CSCs (CD133^+^ CD44^+^ CD24^+^ ESA^+^) and Kras^G12D transgenic mouse model; in vitro human primary CSC cultures.	[196]
EGCG (epigallocatechin-3-gallate)	ALDH^+^ compartment reduction; tumorsphere inhibition; orthotopic tumor growth from ALDH^+^ cells	EGCG decreased ALDH^+^ CSC tumor growth in orthotopic mouse models and reduced sphere formation in vitro at commonly reported 5–50 µM concentrations; in vivo studies demonstrated reduced growth of ALDH^+^-derived tumors.	SUM-149 (inflammatory breast cancer ALDH^+^ cells), other breast CSC models; orthotopic xenografts.	[197]
Genistein (isoflavone)	Sphere formation; ALDH and CD marker changes; xenograft tumorigenicity	Genistein reduced CSC fraction and sphere formation in breast models; concentrations used in vitro generally ≈10–50 µM, with decreased tumorigenicity in xenografts following pre-treatment or co-treatment in several studies.	MCF-7 and other breast cancer cell lines; in vivo xenograft models reported in primary work.	[135]
Quercetin (flavonol)	Sphere formation; CD44^+^/CD24^−^ fraction; ALDH expression	Quercetin suppressed breast CSC self-renewal and reduced the CD44^+^/CD24^−^ population; experimental concentrations commonly 10–50 µM showed marked inhibition of sphere formation and stemness markers.	Breast cancer cell lines (MCF-7/other lines) and in vitro CSC assays; some in vivo corroboration in preclinical studies.	[198,199]
Parthenolide (sesquiterpene lactone)	Side-population/ALDH; sphere formation (melanospheres)	Very potent in CSC assays: example—complete abolition of melanospheres at 5 µM in melanoma CSC reports; robust depletion of side-population/ALDH^+^ fractions at low micromolar doses in multiple models.	Melanoma CSCs, nasopharyngeal carcinoma CSCs, leukemia and solid-tumor CSC models (in vitro); some clinical phase I/II safety data exist.	[138]
Berberine (isoquinoline alkaloid)	Sphere formation; migration/invasion; ALDH; xenograft tumorigenicity/metastasis assays	Berberine reduced sphere formation, decreased ALDH^+^ and migration/invasion in vitro at micromolar concentrations (≈10–50 µM) and reduced tumorigenicity/metastasis in several animal studies (dosing varied by model).	Colorectal, breast, ovarian, and oral cancer cell models; in vivo xenograft/metastasis models reported.	[192,200,201]

**Table 5:** Clinical and translational evidence summary.

Compound	Trial ID/Study Design (Phase, *n*)	Primary Outcome	CSC-Related Endpoint (If Any)	Result/Status	References
Curcumin (C3-complex) + FOLFOX (CUFOX)	NCT01490996—Phase I dose-escalation → Phase IIa randomized; *n* ≈ (phase IIa subset reported); CUFOX protocol (metastatic colorectal liver metastases).	Safety/tolerability and feasibility of combining oral curcumin with standard FOLFOX chemotherapy; recommended dose for phase II.	None reported (no dedicated CSC biomarker endpoint listed).	Completed—published: phase IIa data indicate curcumsin is safe/tolerable as adjunct to FOLFOX; further study recommended.	[218,219]
AXP107-11 (genistein crystalline) ± Gemcitabine	NCT01182246—Phase I/IB dose-escalation and phase IIa; *n* = 44 (reported phase I cohort).	Determine safety/MTD, PK and preliminary efficacy (objective response rate) of AXP107-11 alone and with gemcitabine in pancreatic cancer.	None reported (no explicit CSC assay endpoints in registry).	Completed/published—Phase I reported acceptable safety and favorable PK supporting further study.	[160,220]
Sulforaphane (broccoli-sprout derived)—early-phase chemosensitizer	NCT03934905—Early-phase/safety study in breast cancer patients receiving doxorubicin (pilot design; small *n*).	Primary: safety of SFN administration with DOX; secondary: exploratory molecular markers.	Exploratory molecular endpoints possible (biomarker panels), but no standardized CSC endpoint required in registry entry.	Recruiting/early-phase (protocol posted)—trial designed to test safety and biomarker effects.	[113,209,221]
Resveratrol (SRT501/micronized resveratrol)—colorectal cancer	NCT00433576—Phase I study (patients with resectable colorectal cancer); *n* small (phase I).	Safety, PK and tissue pharmacodynamics (resveratrol and metabolites in resected tissue).	None reported (no CSC assays listed); tissue PD measurements (Notch1 and other pathway markers in some studies).	Completed—tissue PD and safety data published from small cohorts.	[222,223]
EGCG/Green tea extract—oncology supportive/therapeutic trials	NCT01317953—Oral green tea extract in Small-Cell Lung Cancer (SCLC); Phase and *n* per record (early phase).	Safety, tolerability and preliminary efficacy/supportive outcomes.	None reported (no dedicated CSC endpoints listed in registry).	Completed/varied status across EGCG trials—mixed results; most trials focused on safety/PD rather than direct CSC readouts.	[224,225]
Berberine hydrochloride—prevention of colorectal adenoma recurrence	NCT02226185—Multicenter, randomized, double-blind, placebo-controlled; *n* = 1108 randomized (553 vs. 555) reported in publication.	Primary: recurrence rate of colorectal adenoma after polypectomy (efficacy for chemoprevention).	None reported (no CSC assays listed); endpoint is clinical adenoma recurrence.	Completed—published result: reduced adenoma recurrence with berberine vs. placebo (statistically significant). Follow-up/NCT03281096 ongoing for Phase 2/3 expansion.	[226,227]

## Data Availability

No new data were created or analyzed in this study.

## List of Abbreviations

CSC, Cancer Stem Cell; EMT, Epithelial–Mesenchymal Transition; Wnt/β-catenin, Wingless/β-Catenin Signaling; HH, Hedgehog; SHH, Sonic Hedgehog; PTCH, Patched; SMO, Smoothened; GLI, Glioma-Associated Oncogene; PI3K/Akt/mTOR, Phosphoinositide-3-Kinase/Protein Kinase B/Mammalian Target of Rapamycin; PTEN, Phosphatase and Tensin Homolog; GSK-3β, Glycogen Synthase Kinase-3 Beta; JAK/STAT, Janus Kinase/Signal Transducer and Activator of Transcription; IL-6, Interleukin-6; NF-κB, Nuclear Factor Kappa-Light-Chain-Enhancer of Activated B Cells; ABC, ATP-Binding Cassette Transporters; ABCB1/MDR1, Multidrug Resistance Protein 1; ABCG2/BCRP, Breast Cancer Resistance Protein; ROS, Reactive Oxygen Species; HIF-1α, Hypoxia-Inducible Factor-1 Alpha; TGF-β, Transforming Growth Factor Beta; CXCL12, C-X-C Motif Chemokine Ligand 12; ALDH, Aldehyde Dehydrogenase; PDI, Polydispersity Index; PK, Pharmacokinetics; AUC, Area Under the Curve; NP/NPs, Nanoparticle/Nanoparticles; SLNs, Solid Lipid Nanoparticles; NLCs, Nanostructured Lipid Carriers; MSV, Multistage Vector; ESTA, E-Selectin Thioaptamer; HA, Hyaluronic Acid; LSC/LSCs, Leukemia Stem Cell/Leukemia Stem Cells; BM, Bone Marrow; TNBC, Triple-Negative Breast Cancer; IC_50_, Half-Maximal Inhibitory Concentration; EGCG, Epigallocatechin-3-Gallate; SFN, Sulforaphane; WA, Withaferin A; EpCAM, Epithelial Cell Adhesion Molecule; CXCR4, C-X-C Chemokine Receptor Type 4; MUC1, Mucin 1; SCLC, Small-Cell Lung Cancer; miRNA/miR, MicroRNA; PD, Pharmacodynamic.
